# Molecular modelling of the FOXO4-TP53 interaction to design senolytic peptides for the elimination of senescent cancer cells

**DOI:** 10.1016/j.ebiom.2021.103646

**Published:** 2021-10-21

**Authors:** Hillary H. Le, Suleyman S. Cinaroglu, Elise C. Manalo, Aysegul Ors, Michelle M. Gomes, Burcin Duan Sahbaz, Karla Bonic, Carlos A. Origel Marmolejo, Arnaud Quentel, Justin S. Plaut, Taryn E. Kawashima, E. Sila Ozdemir, Sanjay V. Malhotra, Yavuz Ahiska, Ugur Sezerman, Gunseli Bayram Akcapinar, Joshua C. Saldivar, Emel Timucin, Jared M. Fischer

**Affiliations:** a Cancer Early Detection Advanced Research Center, Knight Cancer Institute, Oregon Health & Science University, USA; b Institute of Health Sciences, Acibadem Mehmet Ali Aydinlar University, Atasehir Istanbul 34752, Turkey; c Eternans Ltd., UK; d Dept of Bioengineering, University of California San Diego, USA; e Dept of Cell, Developmental and Cancer Biology, Oregon Health & Science University, USA; f School of Medicine, Acibadem Mehmet Ali Aydinlar University, Atasehir Istanbul 34752, Turkey; g Division of Oncological Sciences, Knight Cancer Institute, Oregon Health & Science University, USA; h Dept of Molecular and Medical Genetics, Oregon Health & Science University, USA

**Keywords:** Senolytic, FOXO4, TP53, Cancer

## Abstract

**Background:**

Senescent cells accumulate in tissues over time as part of the natural ageing process and the removal of senescent cells has shown promise for alleviating many different age-related diseases in mice. Cancer is an age-associated disease and there are numerous mechanisms driving cellular senescence in cancer that can be detrimental to recovery. Thus, it would be beneficial to develop a senolytic that acts not only on ageing cells but also senescent cancer cells to prevent cancer recurrence or progression.

**Methods:**

We used molecular modelling to develop a series of rationally designed peptides to mimic and target FOXO4 disrupting the FOXO4-TP53 interaction and releasing TP53 to induce apoptosis. We then tested these peptides as senolytic agents for the elimination of senescent cells both in cell culture and in vivo.

**Findings:**

Here we show that these peptides can act as senolytics for eliminating senescent human cancer cells both in cell culture and in orthotopic mouse models. We then further characterized one peptide, ES2, showing that it disrupts FOXO4-TP53 foci, activates TP53 mediated apoptosis and preferentially binds FOXO4 compared to TP53. Next, we show that intratumoural delivery of ES2 plus a BRAF inhibitor results in a significant increase in apoptosis and a survival advantage in mouse models of melanoma. Finally, we show that repeated systemic delivery of ES2 to older mice results in reduced senescent cell numbers in the liver with minimal toxicity.

**Interpretation:**

Taken together, our results reveal that peptides can be generated to specifically target and eliminate FOXO4+ senescent cancer cells, which has implications for eradicating residual disease and as a combination therapy for frontline treatment of cancer.

**Funding:**

This work was supported by the Cancer Early Detection Advanced Research Center at Oregon Health & Science University.

**Research in context**

**Evidence before this study**

The FOXO4-TP53 interaction was previously found to be important for the induction of senescence. Since FOXO4 is only present in a small fraction of non-senescent adult cells, this therapeutic target is amenable to systemic treatment for the elimination of senescent cells. Recently, it was shown that by targeting TP53 and disrupting the FOXO4-TP53 interaction with a novel peptide, FOXO4-DRI, senescent cells underwent TP53-mediated apoptosis. Importantly, clearance of these senescent cells alleviated symptoms associated with different age-related diseases.

**Added value of this study**

In the current study, we chose to take a different approach by rationally designing a series of peptides to preferentially bind FOXO4 and act as senolytics. We found a series of peptides with different senolytic activities in vitro and in vivo. The best candidate peptide was further characterized to show disruption of FOXO4-TP53 foci, activation of TP53 mediated apoptosis and preferential binding to FOXO4 over TP53. Finally, we used different delivery methods to eliminate early melanomas resulting in increased survival and eliminate age related senescent cells.

**Implications of all the available evidence**

Senescence is correlated with a wide range of age-related diseases and clearance of senescent cells can delay disease onset or formation of disease, including cancer. By rationally designing a senolytic to FOXO4, we showed that we can eliminate senescent cancer cells and use combination treatments for senescence induction and apoptosis of cancer cells resulting in increased survival. Our future goal is to use molecular modeling to develop small molecule senolytics that target FOXO4 and break up the FOXO4-TP53 interaction. A successful senolytic could improve quality of life and reduce age-related diseases.

Alt-text: Unlabelled box

## 1 Introduction

Cells are constantly acquiring DNA damage that is either repaired by DNA repair machinery or, if extensive, may induce apoptosis [1, 2]. However, cells may use an alternative mechanism in response to DNA damage known as senescence [3, 4]. Cellular senescence is a viable but non-proliferative state that is distinct from quiescence and permanent differentiation. Senescence was first described in cell culture when cells would undergo the Hayflick limit and cease to proliferate after a finite number of cell divisions [5]. Senescence has since been identified as the response to a wide number of stressors including genotoxic stress, oncogene activation and physical damage [6]. Senescence is correlated with a wide range of age-related diseases and clearance of senescent cells can delay disease onset or formation of disease, including cancer [7], [8], [9].

Cancer is a devastating disease that kills millions of people each year with 10 million new cases diagnosed each year globally. Cancer onset correlates with age, and senescence plays a role at multiple stages during cancer development. First, when an oncogene is activated in a cell resulting in aberrant proliferation, the cell can undergo oncogene induced senescence (OIS) [10]. This induction leads to cell cycle arrest, which can be overcome by additional mutations in genes such as *TP53* and *CDKN2A* [11]. Second, when a neoplasia has formed, a senescent microenvironment can contribute to tumour progression through the release of vesicles and signalling molecules in a process known as the senescence associated secretory phenotype (SASP) [12]. Third, genotoxic therapies can induce senescence of cancer and normal cells that receive sub-optimal levels of therapy [13]. Finally, targeted therapies such as BRAF^V500E^ inhibitors and CDK4/6 inhibitors induce temporary senescence, which can be reversed after removal of therapy [14, 15]. For these reasons, it would be beneficial to develop a senolytic for the removal of senescent cancer cells.

The FOXO4-TP53 interaction was previously found to be important for the induction of senescence [16]. Baar et. al. showed that by disrupting the FOXO4-TP53 interaction with a novel peptide, FOXO4-DRI, senescent cells underwent TP53-mediated apoptosis [17]. Since FOXO4 is only present in a small fraction of non-senescent adult cells, this therapeutic target is amenable to systemic treatment for the elimination of senescent cells. In their study, FOXO4-DRI was designed to mimic the TP53 binding domain of FOXO4 and therefore bind to TP53 [17]. In the current study, we chose to take a different approach by rationally designing a series of peptides to preferentially bind FOXO4 and act as senolytics. We found a series of peptides with different senolytic activities in vitro and in vivo. The best candidate peptide was further characterized to show destruction of FOXO4-TP53 foci, activation of TP53 mediated apoptosis, and preferential binding to FOXO4 over TP53. Finally, we show that intratumoural delivery of our senolytic and a Braf inhibitor can successfully eliminate early melanomas in a mouse model resulting in increased survival and systemic delivery of our senolytic can eliminate senescent cells in old mice.

## 2 Methods

### 2.1 Molecular modelling

The full-length structure of the Forkhead (FH) domain of human FOXO4 was modelled by I-TASSER [18] using the partial crystal structure of FH (PDB ID: 3L2C) [19] as the template. Conserved region 3 (CR3) of human FOXO4 was similarly modelled by I-TASSER using the NMR structure of the CR3 domain of FOXO3 (PDB ID: 2LQH) [20] as the template. The resulting FH and CR3 domains of the human FOXO4 which spanned the amino acids from 87 to 177 and from 468 to 502 respectively were evaluated by PROCHECK [21] and WHATCHECK [22]. The structure of TP53DBD was directly obtained from the crystal structure of 3KMD [23] in which the structural zinc was kept and only single polypeptide chain was allocated to docking while other chains including DNA were removed.

### 2.2 Protein-protein docking

The complex structures of TP53DBD-CR3 and TP53DBD-FH were modelled in three steps. First, rigid docking was carried out by ClusPro [24]. Top-10 balanced models were visually inspected to determine the binding interface. Next, flexible docking was performed by the expert interface of HADDOCK [25]. The active and passive amino acids required for HADDOCK were obtained from the binding interface predicted by rigid docking. After flexible docking, the complexes were refined by HADDOCK.

### 2.3 Molecular dynamics simulations

Molecular dynamics simulations were performed by Gromacs 5.1.4 [26] adopting the CHARMM36 all atom force field [27]. The modelled peptide/protein structures, either free or complex forms, were solvated by the TIP3P water model [28] by placing them in the centre of a cubic box with a padding distance of at least 12 Å between the macromolecule and the box edge. The solvated systems were neutralized by adding counter ions of Na^+^ and/or Cl^−^. Neutralized systems were subsequently energy-minimized by the steepest descent algorithm with a maximum force of 240 $kcal.mol^{-1}.nm^{-1}$ Equilibration simulations were carried out as NVT ensembles for 100 ps while the production simulations were NPT ensembles and lasted for at least 40 ns. Nose-Hoover [29] and Parrinello-Rahman algorithms [30] were used to maintain temperature at 310 K and pressure at 1 bar respectively. Non-bonded interactions were calculated up to a cut-off of 12 Å. All hydrogen bonds were constrained with a LINear Constraint Solver (LINCS) algorithm [31]. Coulomb interactions were evaluated with the Smooth fast Particle Mesh Ewald (SPME) electrostatics method with an initial short-range cut-off of 12 Å [32]. The leap-frog algorithm was used to integrate the equations of motion for MD simulations with the periodic boundary conditions. An integration time step was set to 2 fs for all simulations. The trajectories were analysed by means of backbone root mean square (rms) displacements and Cα fluctuations, number of intermolecular contacts and atom-atom distances. VMD [33] or UCSF Chimera [34] was used for visualization of the structures and/or trajectories.

### 2.4 Calculation of the relative binding free-energy

The binding free energy ($\Delta G_{bind}$) of protein-peptide complexes was estimated by the molecular mechanics/generalized Born surface area (MM-GBSA) method [35, 36]. DOCK 6.9 tool [37] was implemented to obtain the individual energy components of the equation given below,

$$\Delta G_{bind}=E_{MM}+G_{polar}+G_{nonpolar}-T\Delta S$$

where $E_{MM}$ is the molecular mechanics free energy in the gas phase, including electrostatic and van der Waals contributions. Solvation free energy is calculated from the polar ($G_{polar}$) and nonpolar ($G_{nonpolar}$) contributions. The term, TΔS which was not included in the calculations represents the conformational entropy at the temperature T. At least 20 different complex conformations obtained from the last stable period of production simulations were used for calculations. The parameters of MM-GBSA calculation were adapted from the reference study [38].

### 2.5 Rational design and random selection

Peptiderive of Rossetta [39] was used to derive the initial peptide sequence that can inhibit the CR3-TP53DBD interaction. Both visual inspections and Peptiderive proposed the N-terminal of the FH (PRKGGSRRNAWGNQSYAELISQAIESAPEKRLTLA) as a peptide inhibitor of the CR3-TP53DBD complex. All L-peptide structures were predicted by PEP-FOLD3 [40] and refined similarly by MD simulations. While the primary structure of the FOXO4-DRI was generated by BIOVIA Discovery Studio Visualizer (v17.2.0) and its tertiary structure was obtained after 2 µs of explicit-solvent MD simulation. For docking, 10 different conformations of each peptide and their partner; CR3 or TP53DBD were extracted from MD simulations. These peptide conformations were cross-docked to the CR3 conformations by QuickVina-W (QVina-W) which employs a specialized algorithm for global docking [41]. During docking, peptide side chains were treated as flexible. Top-scoring model for each complex was selected based on QVina-W scores to be subsequently analysed in MD simulations lasted for 40 ns. Lastly, binding free energy of the CR3-peptide complexes was predicted by the MM-GBSA method. Overall, 4 designed peptide sequences were selected for experimental analysis.

Structure-based screening was performed against the CCPsite 2.0 database which contains 1558 cell penetrating peptides (CPP) [42]. Initially, primary structures of peptides were retrieved from the database and their tertiary structures were predicted by PEP-FOLD3 [40] or I-TASSER [43]. This library of CPP structures was docked to 10 different conformers of CR3 by QuickVina-W. Top scoring 23 CPP-CR3 complexes were analyzed by 40 ns of MD simulations and their binding free-energy was subsequently predicted by the MM-GBSA method. Top scoring CPP was recruited to experiments. After testing normality of the binding free energy distributions that were calculated for 10 different CR3 conformers, between-subject ANOVA was used to compare the means of the peptide binding free energies. Post hoc comparisons were conducted by Tukey HSD test. These analyses were performed by R (version 3.6.3) and R Studio (1.1.442). A total of 5 peptides were purchased from Chinese Peptide Company Ltd. (China), New England Peptide (USA), or GenScript (USA) at >95% purity and the fluoroacetate replaced by acetate. Before synthesis, a D-arginine amino acid is inserted to the N-terminus of the designed sequences. Peptides were prepared in aqueous buffer at concentrations up to 30 mg/mL (Phosphate Buffered Saline (PBS) for in vitro experiments and saline for in vivo experiments).

### 2.6 Cell culture

A375-Luc/iRFP cells were purchased from Creative Biogene (CSC-RR0254). B16-F10 cells were purchased (ATCC Cat# CRL-6475, RRID:CVCL_0159). MCF7 cells were purchased (ATCC Cat# HTB-22, RRID:CVCL_0031). IMR90 cells were purchased (ATCC Cat# CCL-186, RRID:CVCL_0347). A375-Luc/iRFP, B16-F10, IMR90, and MCF7 cells were cultured in Dulbecco's Modified Eagle Medium (DMEM), 10% fetal bovine serum (FBS) and 1X penicillin-streptomycin (PS). HCT-116 cells were purchased (ATCC Cat# CCL-247, RRID:CVCL_0291). DLD-1 cells were purchased (ATCC Cat# CCL-221, RRID:CVCL_0248). SW480 cells were purchased (ATCC Cat# CCL-228, RRID:CVCL_0546). HCT-116, DLD-1, and SW480 cells were cultured in Roswell Park Memorial Institute 1640 (RPMI 1640), 10% FBS, and 1X PS. MCF10a cells (ATCC Cat# CRL-10317, RRID:CVCL_0598) and MCF10a *TP53* knockout cells (Horizon Discovery Cat# HD+101-005, RRID:CVCL_JM25) were purchased and cultured in DMEM/F12 supplemented with 10% FBS, 1X PS, horse serum, epidermal growth factor, hydrocortisone, cholera toxin, and insulin (Lonza/Clonetics CC-3150). Cell lines were tested for mycoplasma at different times in 2019 and the results were negative. Human cell line authentication assays were performed in the OHSU DNA Services Core and confirmed their identity on March 27, 2020. The cell lines were cultured for approximately 3 months in between freezing for each set of experiments.

### 2.7 Drug efficacy in culture

Cell lines were treated with chemotherapy, doxorubicin (Sigma, Cat. No. 2252) to induce senescence (200 nM for HCT116, 100 nM for B16-F10 and MCF7, 50 nM for SW480 and DLD-1, 40 nM for A375-Luc/iRFP and MCF10a). MCF7 cells were treated with CDK4/6 inhibitor, palbociclib (Selleck Chemicals: S1116) to induce senescence (5 µM). A375 cells were treated with Braf inhibitor, Dabrafenib (Selleck Chemicals: S2807), to induce senescence (10 µM). Peptide efficacy experiments were performed by seeding 10,000 – 20,000 cells in a 96-well plate. The cells were given doxorubicin twice to make the cells senescent. After removal of doxorubicin, the plate was treated with varying concentrations of peptide. The peptide was replenished daily for two days. Cellular proliferation was quantified using the Promega CellTiter 96™ AQueous One Solution Cell Proliferation Assay (MTS) after 2 hours according to the manufacturer's protocol (Promega, G3581). To control for the charge of ES2, ProteoJuice (Millipore Sigma, 71281) was used to deliver ES2 using the manufacturers protocol, except 50 µL of 2 mM ES2 was mixed with 25 µL of ProteoJuice. Varying concentrations of the mixture were administered to A375 cells along with a control of only ProteoJuice.

### 2.8 Senescence Associated β-galactosidase staining

Senescent and dividing cells or tissue sections were fixed with 4% formaldehyde. The cells were washed with PBS twice before staining with X-gal solution [1 mg/mL 5-bromo-4-chloro-3inolyl-β-D-galactoside (Boehringer Manneheim #745-740) in N,N-dimethylformamide, 5 mM potassium ferricyanide crystalline (#P-8131), 5 mM potassium ferricyanide trihydrate (#P-3289), 2 mM magnesium chloride, and 1x PBS, pH 6.0. Cells or tissue sections were incubated at 37°C for 24 hours. After staining, cells were washed with PBS before being counted and imaged on the Leica Dmi8 microscope.

### 2.9 Immunofluorescence staining

For all immunofluorescence experiments, cells were plated in 96-well plates (Cellvis P96-1.5P). At the end of each experiment, cells were washed once with PBS and then fixed with 4% paraformaldehyde for 10 minutes at room temperature. For experiments with caspase-3/7 staining, the Caspase-3/7 reagent (Thermo Scientific Cat# C10723) was added to live cells for 30 minutes prior to fixation. Fixed cells were washed with PBS and then permeabilized with ice-cold methanol at 4°C for 10 minutes. Cells were washed with PBS and then blocked for 30 minutes with 1% Bovine Serum Albumin (BSA) in PBS. Cells were stained overnight at 4°C with the following primary antibodies diluted in 1% BSA in PBS: FOXO4 (Cell Signaling Technology Cat# 9472, RRID:AB_10831833, diluted 1:500), CDKN1A (Cell Signaling Technology Cat# 2946, RRID:AB_2260325, diluted 1:500), CDKN2A (Abcam Cat# ab108349, RRID:AB_10858268, diluted 1:500), TP53 (Cell Signaling Technology Cat# 9282, RRID:AB_331476, diluted 1:500), and TP53BP1 (BD Biosciences Cat# 612523, RRID:AB_399824, diluted 1:500). Primary antibodies were removed and cells were washed with PBS. Cells were stained with secondary antibodies (Goat anti-Mouse AF488, Thermo Fisher Scientific Cat# A-11001, RRID:AB_2534069; Goat Anti-Mouse CF 568, Biotium Cat# 20301, RRID:AB_2819163; Goat anti-Rabbit AF 488, Thermo Fisher Scientific Cat# A-11034, RRID:AB_2576217; Goat anti-Rabbit AF 647, Thermo Fisher Scientific Cat# A-21244, RRID:AB_2535812) diluted 1:1000 in 1% BSA in PBS and counterstained with DAPI (5 µg/ml) for 1 hour at room temperature. Cells were washed and stored in PBS until imaged.

### 2.10 Quantitative image-based cytometry

Quantitative imaging was performed as described [44] with the following exceptions. Images were captured on a fully-automated Zeiss AxioObserver with Definite Focus at Oregon Health and Science University's Advanced Light Microscopy Core. The images were captured with a Hamamatsu Orca-flash 4.0 LT Camera using a Plan-Apochromat 20x NA 0.8 objective. Quantification of fluorescence intensities was performed using CellProfiler. Briefly, the DAPI staining was used to create a nuclear mask, and fluorescence intensities of the stained markers (FOXO4, CDKN2A, CDKN1A, and TP53) and FOXO4 foci were measured within the nuclear mask. DAPI fluorescence intensity was also quantified and used for DNA content measurements. Cytometry scatterplots were generated using Tibco Spotfire. For labelling ES2, a cysteine was added to the N terminus and BODIPY FL maleimide (ThermoFisher, B10250) was used to conjugate the fluorophore to the peptide following the manufacturer's protocol. ES2 was incubated with the A375 cells for 2 hours at varying concentrations. Images of ES2 and FOXO4 were captured with a Yokogawa CSU-X1 on Zeiss AxioObserver spinning disk confocal using a Plan_Apochromat 63x oil, NA 1.4 objective.

### 2.11 Proximity Ligation Assay

Senescent and dividing A375 cells were plated in 96-well plates (Cellvis P96-1.5P) at concentration of 20,000 cells per well. The following day, the cells were treated with 8 µM ES2 for 0, 1, and 4 hours. The plate was then fixed with 4% paraformaldehyde for 10 minutes at room temperature. Cells were washed with PBS twice then permeabilized with 0.1% Triton™ X-100 in PBS for 15 minutes. The duolink® In Situ Red Starter Kit Mouse/Rabbit was then used according to manufacturer's protocol with exception of 1:10 dilution of PLUS and MINUS PLA probes. Primary antibodies used were FOXO4 (Cell Signaling Technology Cat# 9472, RRID:AB_10831833, diluted 1:1000), TP53BP1 (BD Biosciences Cat# 612523, RRID:AB_399824, diluted 1:2000), and TP53 (Santa Cruz Cat# sc-126, RRID:AB_628082, diluted 1:500). Z-stacks with 0.34 um steps were captured on a Zeiss Axio Observer Z.1 inverted microscope using a Plan-Apochromat 40 × 0.95 NA lens. The system is setup for widefield fluorescence imaging using a Colibri 7 LED source and a Hamamatsu Orca-flash 4.0 LT black and white camera. The widefield z-stacks were flattened into maximum intensity projections as a pre-processing step before the analysis. Fluorescent foci were counted using the Zeiss Zen software Analysis feature. Briefly, the DAPI staining was used to define the nuclear regions. Within each nuclear region PLA foci were defined by intensity thresholding, and objects were separated by watershed segmentation. The total number of foci per nucleus was recorded.

### 2.12 Co-immunoprecipitation of Myc-FOXO4 for p53

HCT116 cells were transduced with FOXO4 (NM_005938) human tagged ORF Lentiviral Particles (Origene, Cat# RC213185L1V) at 10^6 TU/mL. Cells were then cultured for seven passages. HCT116-Myc-FOXO4 were made senescent using 200 nM Doxorubicin at the final cell concentration of 24 million cells. Dividing cells were plated at concentration of 80 million cells. The following day, cells were treated with 8 µM ES2 for 0 or 1 hour. Nuclear protein was then isolated using NE-PER™ Nuclear and Cytoplasmic Extraction Reagents kit (Cat# 78833) then quantified via Pierce^TM^ BCA Protein Assay Kit (Cat# 23225) according to manufacturer's instructions. 400 µg of each nuclear lysate and 10 µL of provided positive control was used in the Thermo Scientific™ Pierce™ c-Myc-Tag IP/Co-IP Kit (Cat# 23620). The eluted protein (20 µL) was then loaded onto Bolt™ 4 to 12%, Bis-Tris, 1.0 mm, Mini Protein Gel, 10-well (Cat# NW04120BOX) and run at 120V for 60 minutes in 1X Invitrogen™ Bolt™ MOPS SDS Running Buffer using the Mini Gel Tank (Cat. A25977). Protein was then transferred onto 0.4 µm PVDF according to manufacturer protocol. The membrane was then blocked in 5% non-fat milk in 1x TBS-T (Cat# 03-500-537) for 60 minutes. The p53 antibody cocktail (Thermo Fisher Scientific Cat# MA5-14067, RRID:AB_10981528) was then diluted at 1 µg/mL in blocking solution and incubated with the membrane on a slow rocker at 4°C overnight. The membrane was then washed with 1x TBS-T on a rocker for a total of 30 minutes at intervals of 5, 5, 10 and 10 minutes. A secondary antibody of anti-mouse IgG, HRP-linked antibody (Cell Signaling Cat# 7076, RRID:AB_330924) was added to the membrane at dilution of 1:1000 for 45 minutes at room temperature on a slow rocker. The membrane was washed again then imaged on the iBright Imaging System after adding Thermo Scientific™ SuperSignal™ West Pico PLUS Chemiluminescent Substrate for 5 minutes (Cat. 34577).

### 2.13 ddPCR

Primers and probes were designed specifically for ddPCR for human *BBC3* (Bio-Rad Cat# 10031255, Assay ID dHsaCPE5034069) and *PMAIP1*(Bio-Rad Cat# 10031252, Assay ID dHsa5037552) genes. RNA was extracted using the Qiagen RNeasy Kit (Cat# 74004). 5 ng of RNA was used for 22 µl PCR reaction mixtures using the One-Step RT-ddPCR Advanced Kit for Probes (Bio-Rad Cat# 1864022) following the manufacturer's protocol. ddPCR droplets were generated using the Bio-Rad Automated Droplet Generator. Plate containing ddPCR droplets was sealed and RT-PCR was performed using the C-1000 Thermal Cycler (Bio-Rad). ddPCR was performed in the QX200 Droplet Digital PCR System (Bio-Rad). Analysis of the ddPCR data was performed using QX200 analysis software.

### 2.14 Bio-layer Interferometry assay

Bio-layer Interferometry (BLI) measurements were performed on a ForteBio Octet RED384 instrument (Molecular Devices) using ForteBio Data Acquisition software 9.0 and ForteBio biosensors (Molecular Devices) according to the manufacturer's instructions. Kinetic assays were carried out at 30°C using settings of Standard Kinetics Acquisition rate (5.0 Hz, averaging by 20) at a sample plate shake speed of 1,000 rpm. Streptavidin sensors (SA) (ForteBio) equilibrated in PBS were mock loaded (no ligand reference sensor) or loaded with 50 nM of N- terminal biotinylated ES2 peptide (New England Peptide, Inc.) with an approximate response of 1 nm. Ligand loaded SA sensors equilibrated in Kinetics buffer (ForteBio) to establish a baseline, were dipped into wells containing 2-fold dilutions of TP53 (Millipore Sigma Aldrich; Cat# 23-034), FOXO4 (OriGene Technologies; Cat# TP760655) or GFP analytes in kinetics buffer, or buffer alone (reference sample). Increasing concentrations of analytes ranging from 6.2 - 200 nM were allowed to associate for 240 secs, followed by 600 sec dissociation step where the TP53/FOXO4-bound sensors were washed with kinetics buffer. Binding curves were analysed using ForteBio Data Analysis HT 10.0 evaluation software. To control for background signal and non-specific binding, raw experimental data were processed by subtracting reference biosensor and reference sample (no ligand). Processed data from at least six different concentrations of analyte binding were globally fit to a 1:1 Langmuir binding model to calculate the association rate constant *k*_a_*,* the dissociation rate constant *k*_d_ to achieve R^2^ > 0.90. Equilibrium dissociation constants *K*_D_ which defines the strength of the interaction or affinity was calculated as the kinetic dissociation rate constant divided by the kinetic association rate constant.

### 2.15 Circular dichroism and secondary structure estimation

For secondary structure estimation and analysis, circular dichroism (CD) spectra of ES2 were acquired with a JASCO J-1500 spectropolarimeter (JASCO Inc., Easton, MD) using closed, demountable far-UV quartz cuvettes with 0.1 mm path lengths (Starna 20/O-Q-0.1). ES2 was prepared at 1.67 mg/ml in dilute low UV-absorbance CD buffer (1 mM NaH_2_PO_4_, 13.7 mM NaF, 0.3 mM KF, adjusted to pH 7.4 with NaOH and 0.1 µm filtered) with or without 50% 2,2,2-trifluoroethanol (TFE). TFE is a nanocrowding agent that reveals the propensity of peptides to form alpha-helices and it is used here to mimic a crowded binding environment [45], [46], [47]. Spectra were acquired at 37°C, 21°C, and 4°C from 185 to 260 nm at 20 nm/min, 1 nm bandwidth, 2 s integration time, and 0.5 nm data pitch. Lower temperatures were used to reduce thermal motion of ES2 molecules to further reveal helix-forming propensity. For each condition, a minimum of 5 spectra were averaged and background corrected using the average of 3 cuvette- and buffer-matched spectra. The data were then smoothed using a Savitzky-Golay filter and converted to units of molar CD, Δε (M^−1^ cm^−1^). Secondary structural analysis was performed on the spectra of ES2 in CD buffer with and without 50% TFE at 4°C using the DichroWeb online CD data analysis server [48, 49]. To estimate secondary structural content, the CDSSTR program [50], [51], [52] was used with DichroWeb reference dataset 6, which is a 42 protein set including denatured proteins that increase coverage of the structural space occupied by peptides [50]. Normalized root-mean-square deviation of the reconstructed data was <0.025 for both analyses, which suggests that the reference dataset and model are appropriate for the ES2 data.

### 2.16 Mice

Mice were aged between 3-8 months and 20-30 g in weight and were housed in specific pathogen free cages. Animals were randomly distributed among treatment groups by assigning mice within the same cage to different treatment regimens. To minimise confounders, mouse treatment order was randomised within each cage. Male and female mice were distributed between all treatment groups since the chemicals used were directly injected and are not metabolized. Researchers were not blinded to the group allocation, but all attempts were made to analyse the data without knowing treatment conditions. NOD.Cg-Prkdcscid Il2rgtm1Wjl/SzJ (NSG) were purchased from The Jackson Laboratory (IMSR Cat# JAX:005557, RRID:IMSR_JAX:005557). NOD.Cg-KitW-41J Tyr + Prkdcscid Il2rgtm1Wjl/ThomJ (NBSGW) were purchased from The Jackson Laboratory (IMSR Cat# JAX:026622, RRID:IMSR_JAX:026622). Immunocompromised mice used for these experiments were all in the NSG background; however, some mice were F1 generation of NSG x NBSGW, thus the brown coat colour as NBSGW mice have tyrosinase. Xenografts containing senescent cells were established by injecting A375-Luc/iRFP human melanoma cells which contained a luciferase expressing gene into immunocompromised NSG ears. Senescence was induced in the cells via doxorubicin treatment (40 nM) then resuspended in DMEM prior to injection. Each ear was then injected with 50 µL containing approximately 1,000,000 cells. The ears were treated once daily for two days with 50 µL of either 5 mg/mL peptide, or saline. To control for confounders, each mouse contained one ear treated with saline (control). For imaging, mice were injected with 3 mg luciferin intravenously and imaged before and after treatment. Transgenic mice *(BrafCA, PtenloxP, Tyr::CreERT2*) were purchased from The Jackson Laboratory (IMSR Cat# JAX:013590, RRID:IMSR_JAX:013590). These mice were maintained on a C57Bl6 background and all experimental mice contained *Tyr::CreER* with either *Braf^CA/+^, Pten^fx/+^; Braf^CA/CA^, Pten^fx/+^*, or *Braf^CA/+^, Pten^fx/fx^*. Melanomas were sporadically induced by painting the mouse ears with 4-hydroxytamoxifen (∼25 µL of 30 mg/mL) (Sigma H7904). Hypopigmented melanomas sporadically formed over the next 3-5 months, typically on the back of the mouse. These melanomas were then directly injected with 100 µL of either 20 mg/mL ES2+10%DMSO, saline+10%DMSO, saline+10%Dabrafenib, or 20 mg/mL ES2+10%Dabrafenib. The researchers were not blinded to the experiment, but each mouse contained an internal control and all imaging was done with the same exposure times based upon control tissue. For survival analyses, either 750,000 or 25,000 A375-Luc/iRFP cells were injected intradermally into NSG mice. Different combinations of drugs were locally injected to the region once daily for two days with 50 µL of either 5 mg/mL ES2, 10%Dabrafenib, or 5mg/mL ES2+10%Dabrafenib. Dabrafenib was made at 100 mg/mL in DMSO. Mice were then monitored daily and allowed to age until a humane endpoint was reached according to our IACUC protocol, which in this case was tumours of 2 cm in size. Aged C57Bl6 male mice were purchased from the Jackson Laboratory (IMSR Car# JAX:000664, RRID:IMSR_JAX:000664) at 72 weeks of age and were started on the experiment at 82 weeks. Mice were treated with 3 mg of ES2 via tail vein injection weekly for three weeks and weighed weekly. Blood was drawn prior to beginning experiments and at the endpoint. To generate plasma, blood was centrifuged at 1,500g for 10 minutes, then 2,500g for 10 minutes, then frozen for later analyses. A total of 154 mice were used for these experiments and all animals were included in the experiment until either the end of the experiment or at a humane endpoint based upon our IACUC protocol.

### 2.17 TUNEL staining

Fixed frozen sections of melanomas were stained with the Click-IT Plus TUNEL assay (ThermoFisher C10618) according to the manufacturer's protocol. Stained sections were imaged with a Zeiss A2 upright microscope at either 20x or 10x. At least one of each treatment group were stained and imaged at the same time. All sections from each staining group were set to the same exposure time for TUNEL and DAPI. Any normalization of background levels was performed in Adobe Photoshop and set to the same scale for the entire image and for all other images.

### 2.18 Blood analyses

For complete blood counts, 10 µL of whole blood was mixed with 10 µL of 2 mM EDTA and counts were performed using the HemaVet 950FS (Drew Scientific). The Urea Assay Kit (Abnova Cat# KA1652) was used to measure plasma urea following the manufacturer's protocol.

### 2.19 Ethics statement

All experiments were approved by Oregon Health and Science University (OHSU) Institutional Animal Care and Use Committee (IACUC) (TR01_IP00000674) and conformed to the guidelines set by United States Animal Welfare Act and the National Institutes of Health.

### 2.20 Statistics

Statistics were performed using StatistiXL in Microsoft Excel and SPSS from IBM. Tests of normality (Shapiro-Wilk) were performed to determine if the data were normal and could be analysed with parametric tests. For data with two variables, the student T-test was used. For data with more than 2 variables, ANOVA was used followed by the Scheffe or Tukey post-hoc test. Kruskal-Wallis was also used followed by Bonferonni correction. Survival data were analysed using the Log Rank test. Annexin V slope data were analysed with linear regression comparison. No specific protocol was prepared before the study. Study data can be obtained from the corresponding author.

### 2.21 Role of funders

This work was supported by the Cancer Early Detection Advanced Research Center at Oregon Health & Science University. The funders had no role in the experimental design, collection of data or writing of the paper.

## 3 Results

### 3.1 Design and selection of peptides targeting CR3

**Fig. 1:**
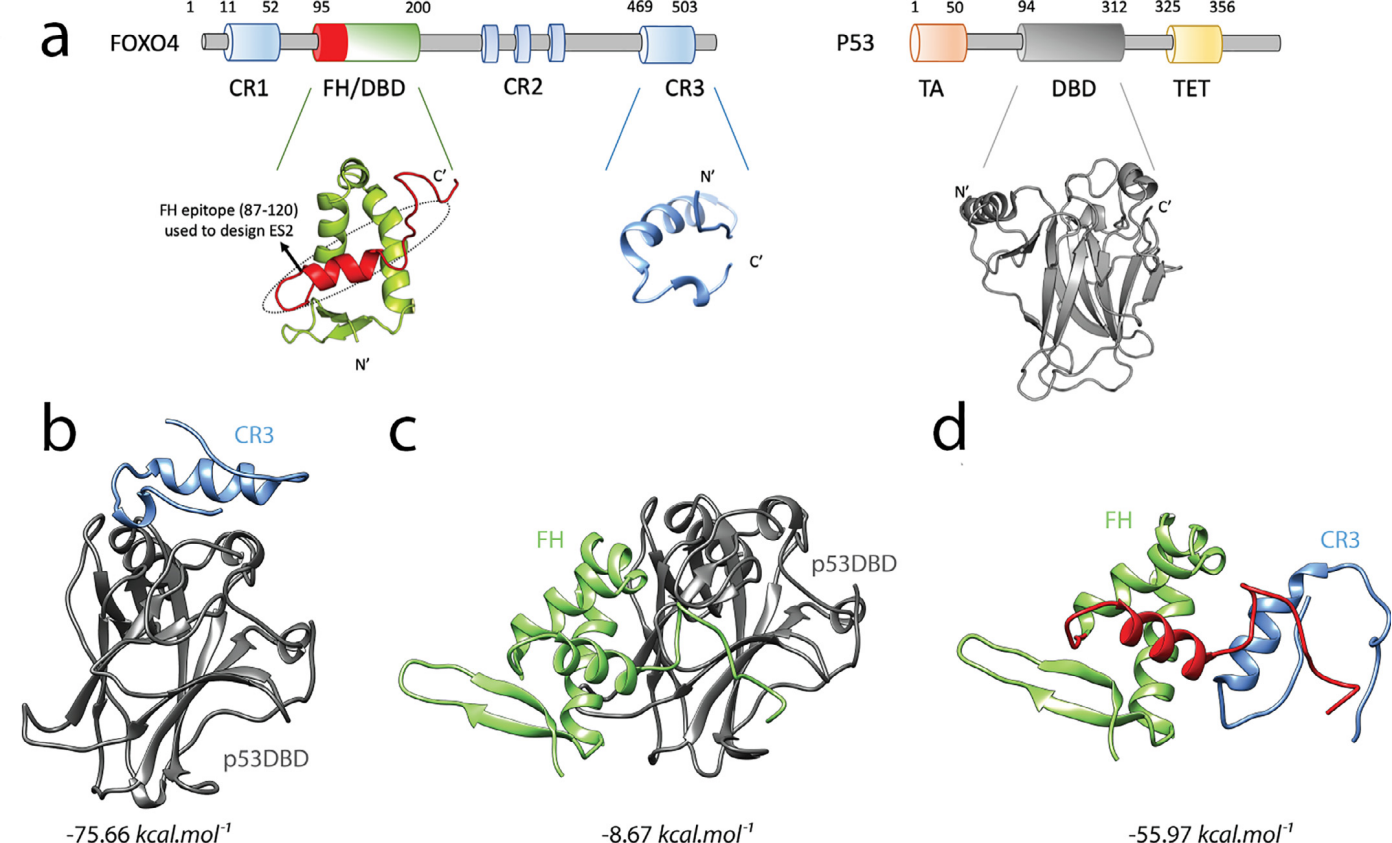
Computational design of peptide targeting FOXO4. **a**, Domains of FOXO4 and TP53 (p53). FOXO4FH (PDB ID: 3L2C), in its closed state, interacts with FOXO4CR3. TP53DBD (PDB ID: 3KMD), disrupts this intramolecular interaction and binds both FOXO4FH and FOXO4CR3. The FH epitope (87-120) which was used to design the senolytic peptide ES2 is coloured red and framed with dashed lines. Protein-protein docking analysis of **b**, FOXO4CR3-TP53DBD, **c**, FOXO4FH-TP53DBD and **d**, FOXO4FH-CR3. The TP53DBD is colored gray, FOXO4FH is green and FOXO4CR3 is blue. The FOXO4 epitope used to design the senolytic peptide ES2 is coloured red. For **b, c** and **d** the binding free energy ($\Delta G_{bind}$) of the complexes which were predicted by MM-GBSA is given. See also Fig. S1 and S2 and Table S1 and S2.

FOXO4 and TP53 are multi-domain proteins that interact with each other and stimulate senescence (Fig. 1**a**). It was previously shown that TP53 disrupts the intramolecular interaction of the Forkhead (FH) domain and the conserved region 3 (CR3) of human FOXO3 (Fig. 1**b-d**) [53]. Moreover, by using FOXO3, the same study showed that FH and TP53 compete for the binding site to the core region of CR3. Because the FOXO family of proteins show functional redundancy and share high sequence identity essentially for the FH and CR domains [54], we note that the binding mechanism previously identified for FOXO3 and TP53 [53] is valid for FOXO4 and TP53. To this end, we used the domain level information reported in the study of Wang et al. to construct the first atomistic model of FOXO4-TP53 and ultimately to design peptides targeting this complex.

Since there is currently no physical structure of the complete FOXO4 protein, the FH and CR3 domains of FOXO4 were individually modelled and their interaction interface was predicted as follows. After addition of the missing ^87^PRKGGS^92^ peptide, the full-length structure of the FH domain was modelled as well as the CR3 domain. Ramachandran plots of the predicted models FH and CR3 domains did not show any violations for the combinations of psi/phi dihedral angles. As such, the FH model has 93.9% (87/93) of its residues in favoured regions, 6.5% (6/93) in generously allowed regions and 0.0% (0/93) in disallowed regions. Similarly, CR3 model similarly showed that 93.5% (33/35) of its residues in favoured regions, 6.5% (2/35) in generously allowed regions and 0.0% (0/35) in disallowed regions. Calculated z-scores for the bond lengths and angles further indicated tight packing of both models, 0.632 and 1.255 for FH and 0.650 and 1.251 for CR3. Overall, the assessment of the structures of the FH and CR3 domains of FOXO4 reflected high quality predictions, validating the use of these models in protein-protein docking studies.

Three different complexes of TP53DBD-CR3 (Fig. 1**b**), TP53DBD-FH (Fig. 1**c**) and FH-CR3 (Fig. 1**d**) were modelled by a series of protein-protein docking calculations. Initially, an FFT-based rigid docking was applied for prediction of the binding interface, then flexible docking and its subsequent refinement was performed. Rigid docking results predicted a well-defined binding interface for the FH-CR3 interaction but not for the TP53DBD-FH interaction and are in agreement with the binding affinity analysis that confirmed much weaker binding affinity for FH to TP53DBD (∼1 mM) compared with its affinity to CR3 (19 µM) [53]. Moreover, the final complexes of TP53DBD-FH (Fig. 1**c**) and TP53DBD-CR3 (Fig. 1**b**) display different binding interfaces on TP53 reflecting the feasibility of a ternary complex of TP53DBD-FH-CR3 which was previously proposed by the NMR shift experiments using TP53 and FOXO3 [53]. Quantitative predictions of binding free energy which sampled 10 distinct complex conformers from MD simulations indicated that CR3 has a comparable binding affinity toward FH and TP53DBD while the complex formed between FH and TP53DBD is much less stable than those formed by CR3 (Fig. 1**b-d**). Overall, these predictions provided us with the first model of the FOXO4-TP53 complex at an atomistic resolution, unravelling the role of the CR3 domain as a transcriptional inhibitor of both TP53 and FOXO4 itself. This inhibitory action of FOXO4 on the transcriptional activity of TP53 could account for the resistance to TP53-mediated apoptosis of senescent cells [55]. Aside from that, these structural models ultimately contributed to the rational design of FOXO4 inhibitory peptides.

Comparative analysis of the predicted structures has led us to identify that the FH domain interacted with the CR3 domain through a continuous epitope (Fig. 1**a and d**), suggesting this epitope as a possible inhibitor for the CR3 domain. This observation was further confirmed by Peptiderive of Rosetta which similarly suggested that the N-terminal region of the FH domain of FOXO4 as a peptide inhibitor of CR3. The first round of rational design was carried out by mutating multiple positions in the native FOXO4 sequence and the resulting peptide was labelled as Eterone 1 (E1). The FOXO4-DRI (DRI) peptide which specifically targets FOXO4-TP53 interaction [17] was included in the analysis as a positive control. Indeed, the first designed sequence E1 contains 4 mutations to the D-retroinverso isoform (**Table S1**). Binding free energy predictions indicated that E1 has a higher affinity toward CR3 than the DRI form (Table 1), confirming our molecular modelling.

**Table 1:** Binding free energy predictions of the complexes.

Complex	$\Delta G_{\mathrm{bind}}$ (kcal.mol^−1^)*
FOXO4FH-CR3	-55.97 ±5.46
FOXO4CR3-TP53DBD	-75.66 ± 7.65
FOXO4FH-TP53	-8.67 ± 6.00
FOXO4DRI-CR3	-106.90 ± 15.13
FOXO4DRI-TP53	-39.92 ± 15.20
E1-CR3	-157.40 ± 6.07
ES2-CR3	-201.33 ± 10.00
ES2r1-CR3	-163.46 ± 7.22
ES2r2-CR3	-155.00 ± 7.30
ES1-CR3	-149.10 ± 9.28
	

⁎ “Mean±SD*”* is given for 10 distinct complex conformers.

**Fig. 2:**
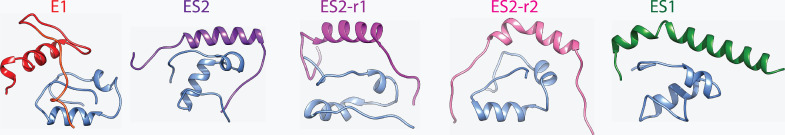
Structures of CR3-peptide complexes.

The binding free energies of DRI toward CR3 and TP53DBD were similarly calculated to be consistent with the binding free energies of the FH domain. As such, either FH domain or FH domain derived peptides such as DRI or E1 show stronger affinity to CR3 than TP53DBD (Table 1). To promote intracellular delivery, both E1 and DRI sequences were fused with the cell penetrating peptide (CPP) sequence of HIV-TAT [56], resulting in a 45-aa long sequence (**Table S1**). However, because the large sizes of E1 and DRI limits drug-likeness properties [57], we carried out another design aiming to curtail E1 sequence while maintaining the same or higher level of CR3 affinity. To carry out this design, we initially decomposed the binding free energy of DRI and observed that the C-terminal tag of the DRI peptide which corresponds to the HIV-TAT tag most likely contributed to the CR3 binding compared with the rest of the DRI sequence (**Fig. S1**). Essentially, the contribution of HIV-TAT to binding free energy (**Fig. S1**) suggests that HIV-TAT sequence does not only facilitate cellular uptake but also involves in targeting to CR3. Hence, in the next round of design, we removed the HIV-TAT and introduced a similar CPP mimicking sequence [58] to the helix which was shown to interact with CR3 (Fig. 2). The resulting peptide sequence was labelled as ES2 (Table 1). To further test the contribution of the arginine repeat, 2 or 4 of the arginines in the ES2 were back-mutated to the native FOXO4 and labelled as ES2r1 and ES2r2, respectively. MM-PBSA calculations reflected that all of the designed peptides showed significantly higher binding free energy toward CR3 than FOXO4-DRI **(Fig. S2)**. Among all peptides, ES2 had the highest CR3 binding free energy and was followed by the ES2r1 while the remaining two peptides; E1 and ES2r2 had comparable affinities towards CR3.

Reinforced by the observation of the strong interaction between the fused CPP and CR3, we also screened a CPP library of 1558 members against 10 different conformations of CR3. Binding free energy predictions strikingly indicated a similar TAT-based CPP as the strongest CR3 binder (**Table S2**). Together with the designed sequences (E1, ES2, ES2r1 and ES2r2), a triple repeat of HIV-TAT, labelled as ES1, was recruited to the same *in silico* workflow, producing a comparable CR3 affinity with E1 (Table 1). Notably, binding free energy of ES1 after screening (Table 1) were in close agreement with that obtained during screening (**Table S2**), reflecting the reproducibility of our MM-GBSA based predictions. Taken together with all of the tested peptides, our computational findings indicated ES2 as the most promising candidate to target CR3, which was followed by the back-mutated r1 and the screening outcome of ES2. On the other hand, as confirmed by the binding free energy predictions showed that the positive control peptide; DRI was the weakest CR3 binder (Table 1 **and Fig. S2**). Overall, computational studies led to identification of 5 distinct L-peptide sequences to inhibit the FOXO4-TP53 complex, reflecting the influence of positively charged amino acids for targeting CR3.

### 3.2 Efficacy of our senolytic peptides on human melanoma cells in culture

**Fig. 3:**
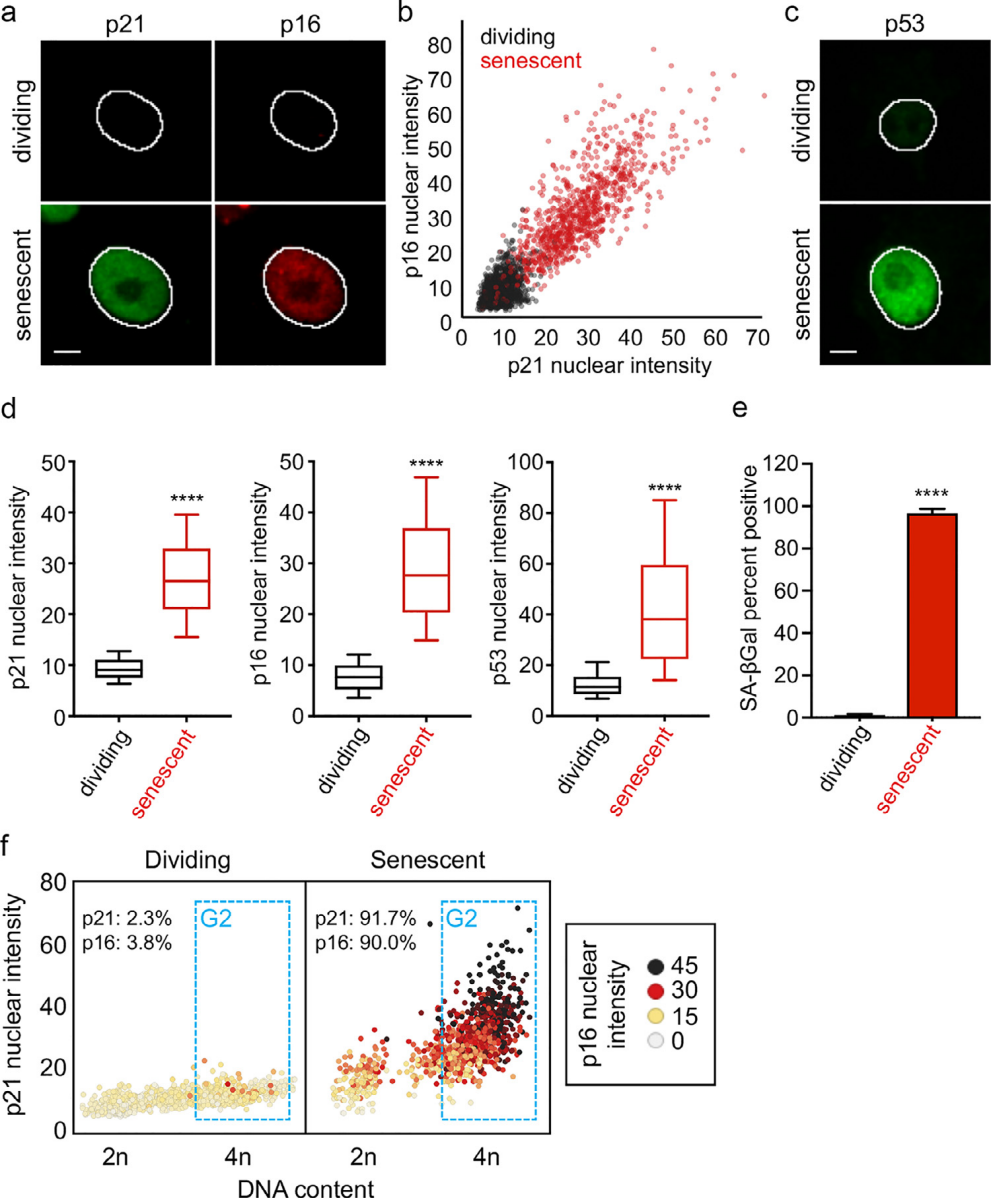
Characterizing senescent cells. **a**, Representative images of CDKN1A (p21) and CDKN2A (p16) in A375 cells. Senescence was induced using doxorubicin (40 nM) for 4 days. The outline of the nucleus is indicated by the white line. Scale bar is 10 µm. **b**, Scatterplot of the p21 nuclear intensity vs. the p16 nuclear intensity of individual cells either mock-treated (dividing) or doxorubicin-treated (senescent) A375 cells. Red dots indicate cells that were treated with doxorubicin. Units of fluorescence intensity are arbitrary. **c**, Representative images of TP53 (p53) in A375 cells treated as described in **a. d**, Box and whisker plots of p21, p16, and p53 nuclear intensities in A375 cells treated as described in **a**. **** p-value < 0.0001. **e**, Bar graph of the percent of A375 cells positive for SA-β-gal. **** p-value < 0.0001. **f**, Scatterplot of the DNA content vs. the p21 nuclear intensity of individual A375 cells treated as in **a**. Color scale indicates the p16 nuclear intensity. Units of fluorescence intensity are arbitrary. The G2 phase cells are gated in blue. Percent positive for p21 and p16 is indicated for both dividing and senescent cells. See also Fig. S3.

**Fig. 4:**
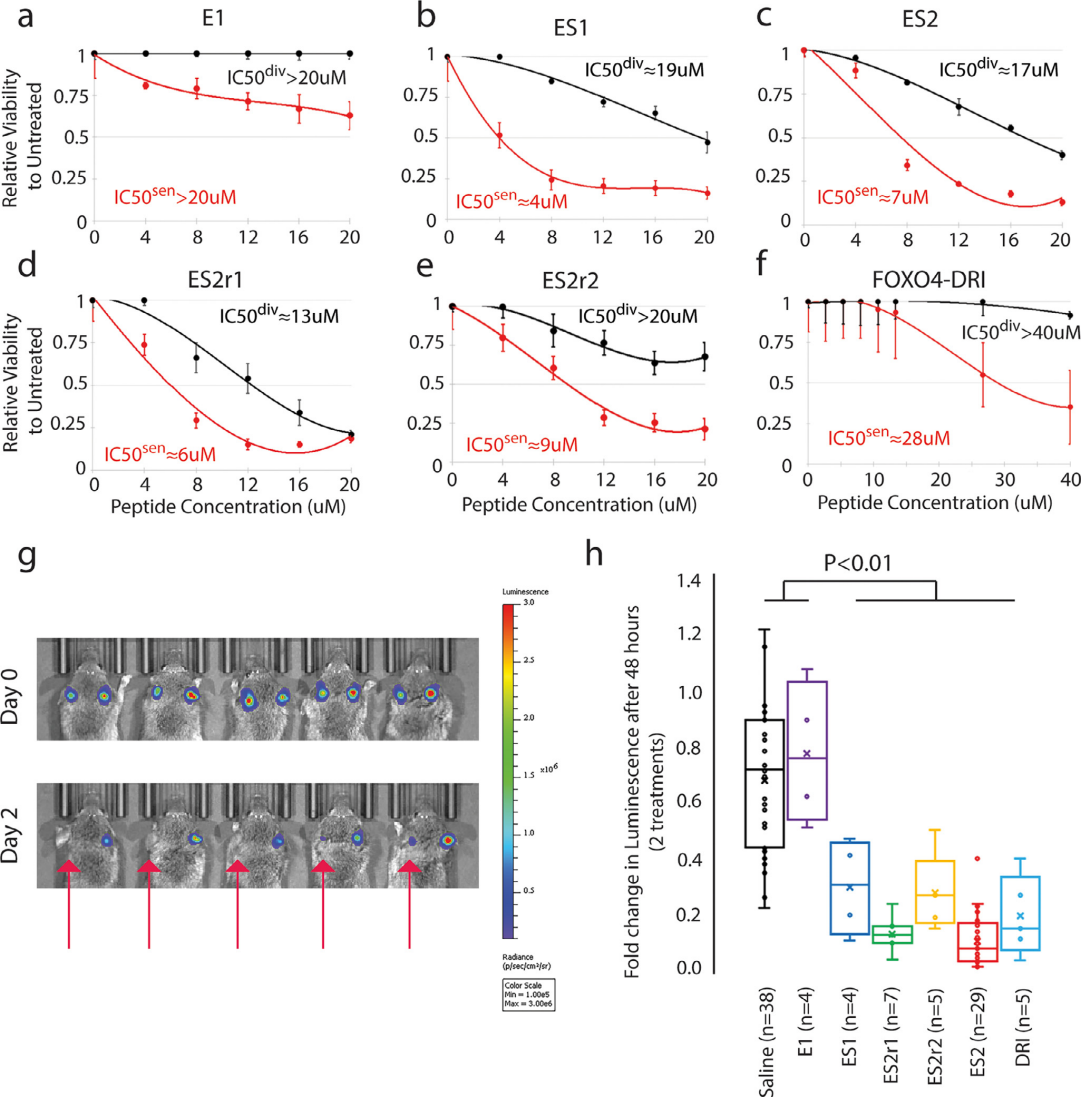
Senolytic activity of a series of rationally designed peptides in cell culture and in vivo. **a-f**, Line plots of the relative viability of the different peptides to dividing (black) or senescent (red) A375 cells: E1 (**a**), ES1 (**b**), ES2 (**c**), ES2r1 (**d**), ES2r2 (**e**) and FOXO4-DRI (**f**). Lines are best fit polynomial. IC50^div^ is the concentration to kill 50% of dividing cells. IC50^sen^ is the concentration to kill 50% of senescent cells. **g**, Images of mice with luciferase expressing, senescent, human melanoma cells injected orthotopically. Both ears of each mouse were injected with an equal number of doxorubicin-induced senescent A375 cells. The left ear was locally injected with ES2 (50 µL of 5 mg/mL) after imaging on day 0 and day 1 (red arrow). The right ear was injected with saline at the same time. Mice were then again imaged at day 2. **h**, Quantification of the fold change in radiance before and after saline or peptide treatment. Since the A375 cancer cells are senescent, there is a slight decrease in signal in the saline treated ears. ES2 (n=29), ES2r1 (n=7), ES2r2 (n=5), ES1 (n=4), and DRI (n=5) are significantly different from saline (n=38) and E1 (n=4) (ANOVA Scheffe post-hoc test). See also Fig. S3.

We first determined the concentration of doxorubicin that induces senescence in A375 melanoma cell line as determined by a block in proliferation and senescence associated beta-galactosidase (SA-β-gal) staining (**Fig. S3a**). In addition, we immunostained for different senescence markers, including CDKN2A (p16), CDKN1A (p21), and TP53 (p53) and measured their abundance in single cells using a quantitative high-content imaging method [44]. Doxorubicin treatment led to a significant increase in these markers, especially in G2 phase arrested cells (p<0.0001) (Fig. 3). Senescent and dividing cells were then treated with different doses of peptides to determine the IC50. We found that ES1 (Fig. 4**b**), ES2 (Fig. 4**c**) and ES2r1 ( Figure 4**d**) has the highest senolytic activity with ES1 and ES2 being comparable in senolytic activity for the difference between dividing and senescent cells at 8 µM. ES2r2 (Fig. 4**e**) had slightly lower senolytic activity and E1 (Fig. 4**a**) had negligible senolytic activity. Finally, we compared our peptides with FOXO4-DRI peptide and found that, while FOXO4-DRI has minimal lytic activity in dividing cells, our peptides had 3-7 times higher senolytic activity (Figure 4f) . These results suggested that there is a therapeutic window for senolytic activity with our modelled peptides.

Since these peptides are highly cationic due to abundance of arginine in the peptide sequence, it is possible that the lytic activity is purely from the cationic nature of the peptide and not the senolytic activity. Therefore, we delivered ES2 with a peptide delivery agent, ProteoJuice, and found no difference in senolytic activity (**Fig. S3c**). Importantly, the highest concentration of ProteoJuice alone showed only 11% cell death (p=0.32), much less than the 80% cell death seen with ES2 (p<0.01). These data suggest that charge alone is not killing the cells in culture, but that apoptosis is specific to an intracellular action of the peptides on senescent cells.

### 3.3 Efficacy of our senolytic peptides on orthotopic human melanoma cells in mice

To determine the efficacy of our peptides on senescent human cancer cells in vivo*,* we utilized an orthotopic xenograft mouse model. A375 human melanoma cells were induced into senescence in culture with doxorubicin. Then these senescent cells were injected intradermally into both ears of immunocompromised NSG mice. To track the cells intravitally, A375 cells contained a Luciferase gene, which has a linear response to the number of cells present [59]. Mice were imaged for luciferase signal 3-7 days after the initial injection for a baseline reading of the number of senescent cells present. To understand the senolytic activity of the peptides, one ear per mouse was locally injected once daily for two days with either peptide or saline. By having internal controls within each mouse (one ear treated with peptide, the other ear with saline), we reduced the variation inherent in delivery of luciferin. The mice were then imaged for luciferase signal 24 hours after the second treatment. We found that ES2, ES2r1, ES2r2, ES1, and DRI had statistically significantly reduced luciferase signal compared to E1 and saline (p<0.01, ANOVA) (Fig. 4**g, h**). For sensitivity analyses, the Kruskal-Wallis test also revealed ES2 to be significantly different from E1 and saline (p<0.002, Bonferroni correction). To further understand the role of ES2 in dividing cells, we injected dividing A375 cells then treated with ES2 and found that it was ineffective at eliminating dividing cancer cells (**Fig. S3b**). Importantly, the peptides followed a similar trend to cell culture for eliminating senescent cancer cells. These results suggested that a subset of our peptides could be used for in vivo elimination of genotoxin-induced, senescent cancer cells with ES2 showing the best senolytic activity in vitro and in vivo.

### 3.4 ES2 preferentially binds and eliminates FOXO4+ cells in a wide range of cell types

**Fig. 5:**
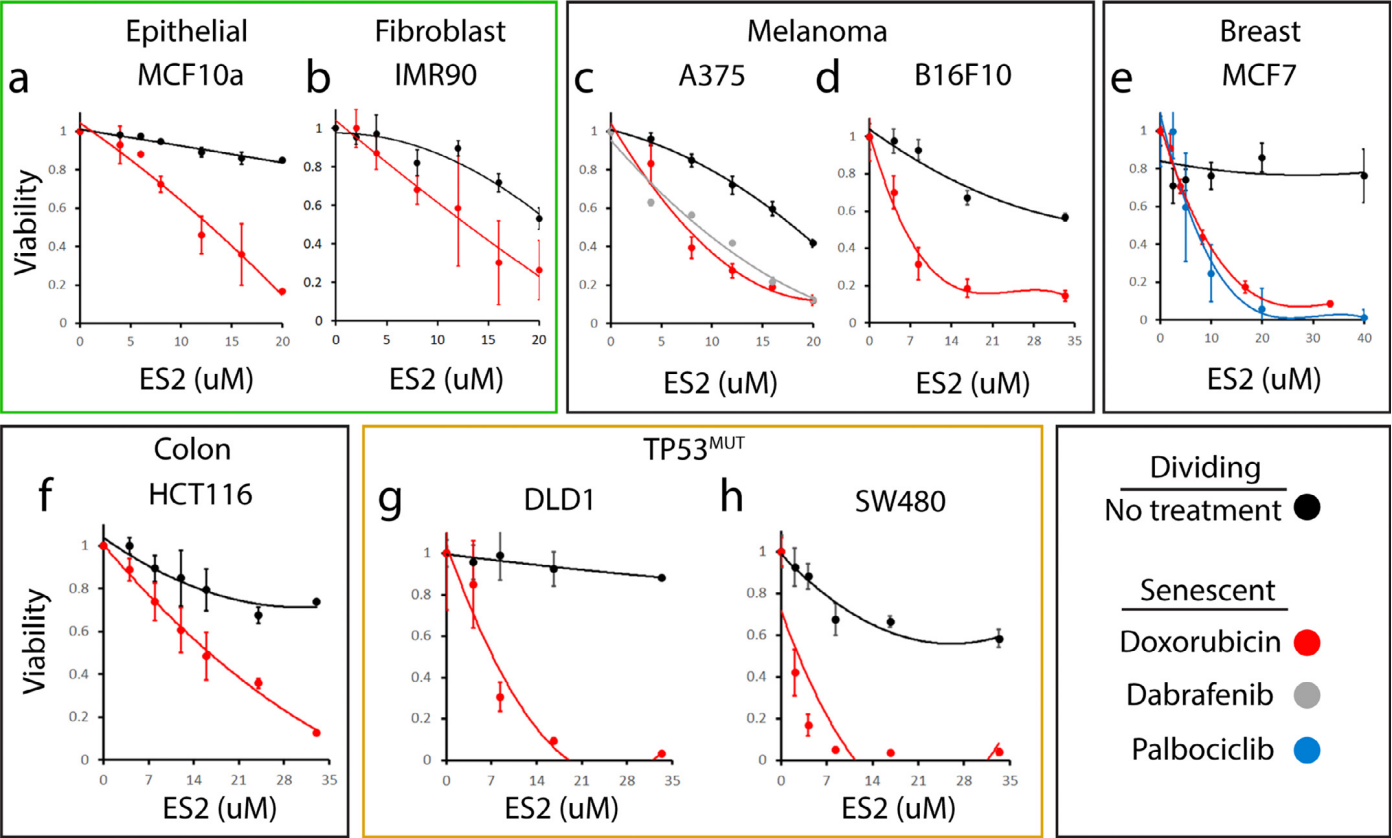
Senolytic activity of ES2 in different cell lines, different senescence inducers and different TP53 status. **a-h**, Line plots of relative viability of seven different cell lines on dividing cells (black) or senescent cells from different senescence inducers: Doxorubicin (red), Dabrafenib (grey), and Palbociclib (blue). Solid lines are best fit polynomial. **a-b,** Normal epithelial cell line, MCF10a, and fibroblast cell line, IMR90. **c-d**, Melanoma cell lines A375 and B16F10 (mouse). **e**, Breast cancer cell line MCF7. **f**, Colorectal cancer cell line HCT116. **g-h,** TP53 mutant colorectal cancer cell lines DLD1 and SW480.

Based on our initial screening, we chose to characterize ES2 at greater depth. To determine the range of senolytic activity for ES2, we used doxorubicin to chemically induce senescence in cells from three different tissues: melanoma (A375 and B16F10), colorectal cancer (HCT116), and breast cancer (MCF7) and normal fibroblasts and epithelium (IMR90 and MCF10A). In all cell lines, the IC50 of ES2 was at least 3 times lower concentration in senescent cells compared to dividing cells suggesting that ES2 increases apoptosis preferentially in senescent cells (Fig. 5). We also tested if the mechanism of senescence induction affects apoptosis caused by ES2. Therefore, we utilized two prosenescent agents, Dabrafenib [60] and Palbociclib [61]. *Braf* mutant A375 cells were treated with Dabrafenib and different concentrations of ES2. *PIK3CA* mutant MCF7 cells were treated with Palbociclib and different concentrations of ES2. We found no difference in concentration of ES2 resulting in cell death when comparing doxorubicin to these pro-senescence agents suggesting that ES2 works robustly on senescent cells regardless of senescence inducer. Finally, we wanted to understand the role of *TP53* mutations on the senolytic activity of ES2. Therefore, we tested two different colorectal cancer cell lines containing different mutations and found that ES2 is effective at inducing cell death in senescent cells (Fig. 5**g, h**). DLD1 is heterozygous for S241F *TP53* mutation, which is a non-functional mutation. SW480 cells are homozygous for two different *TP53* mutations, R273H and P309S. While R273H mutation is non-functional, the P309S mutation is well tolerated [62]. Thus, both cell lines have some functional TP53 suggesting that cancer cells with combinations of mutant and functional *TP53* alleles can still be eliminated by ES2.

**Fig. 6:**
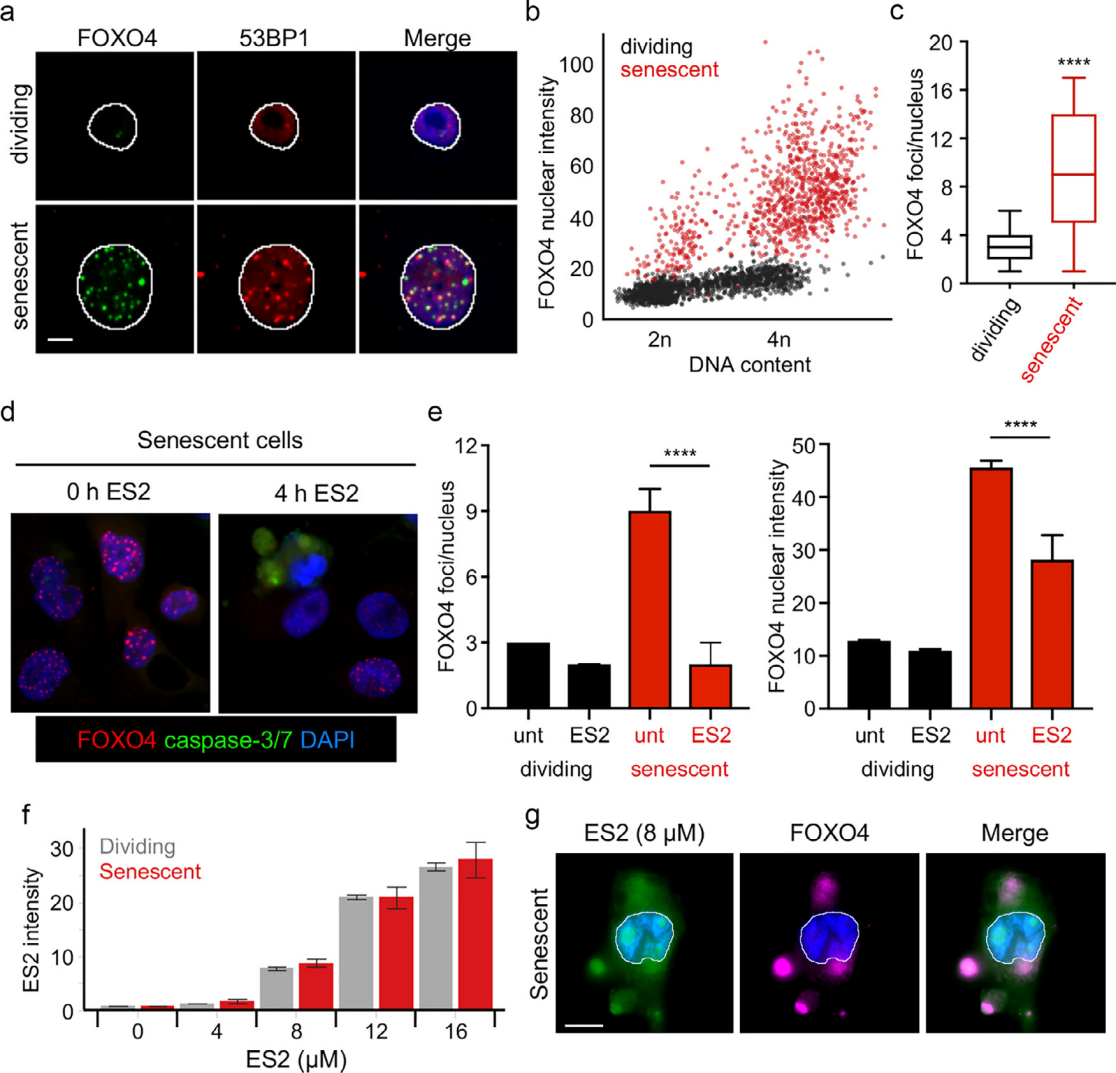
ES2 disrupts FOXO4 foci and localizes to FOXO4 foci**. a**, Representative images of FOXO4 and TP53BP1 (53BP1) in A375 cells treated as described in Fig. 3**a**. Scale bar is 10 µm. **b**, Scatterplot of the FOXO4 nuclear intensity vs. the DNA content (DAPI intensity) of individual cells either mock-treated (dividing) or doxorubicin-treated (senescent) A375 cells. Red dots indicate cells treated with doxorubicin. Units of fluorescence intensity are arbitrary. **c**, Box and whisker plots of the FOXO4 foci/nucleus in A375 cells treated as described in **a**. **** p-value < 0.0001. **d**, Representative images of FOXO4 and active caspase-3/7 in senescent A375 cells treated with ES2 8 µM for 4 h. **e**, Bar graphs of the median FOXO4 foci/nucleus and median FOXO4 nuclear intensity in A375 cells treated as described in **e**. unt (untreated). Error bars denote the 95% confidence interval. **** p-value < 0.0001. **f**, Plots of median fluorescence intensity of ES2-BODIPY at 2 h after addition of labeled ES2 to dividing or senescent A375 cells. **g**, Representative images of A375 senescent cells 2 h after addition of ES2-BODIPY showing the overlay of FOXO4 (purple) and ES2 (green) outside of the nucleus in dying cells. The nucleus is stained with DAPI (blue) and outlined. Scale bar = 10 µm.

Since ES2 was designed to disrupt the FOXO4–TP53 interaction, we measured FOXO4 levels and foci in senescent and dividing cells. Consistent with previous findings [17], FOXO4 levels increased dramatically in senescent cells compared to dividing cells (Fig. 6**a, b**). Furthermore, the number of FOXO4 foci also increased significantly in senescent cells (p<0.0001) (Fig. 6**c**). These foci frequently overlapped with TP53BP1 (53BP1) foci (Fig. 6**a**), suggesting FOXO4 interacted with TP53 as previously described [17]. Importantly, ES2 addition caused a loss of FOXO4 foci shortly prior to activation of caspase-3/7 and apoptosis (Fig. 6**d, e**). Finally, we labelled ES2 with a neutral fluorophore, BODIPY, and found that the ES2 peptide accumulated inside cells in a dose-dependent manner, co-localized with FOXO4, and disrupted focus formation. Importantly, this effect was only observed at concentrations (≥ 8 μM) that induce apoptosis of senescent cells (Fig. 6**f, g**). These results suggest that ES2 disrupts the FOXO4-TP53 interaction to induce apoptosis of FOXO4+ senescent cells.

### 3.5 ES2 rapidly breaks up FOXO4-TP53 foci and activates TP53 mediated apoptosis

**Fig. 7:**
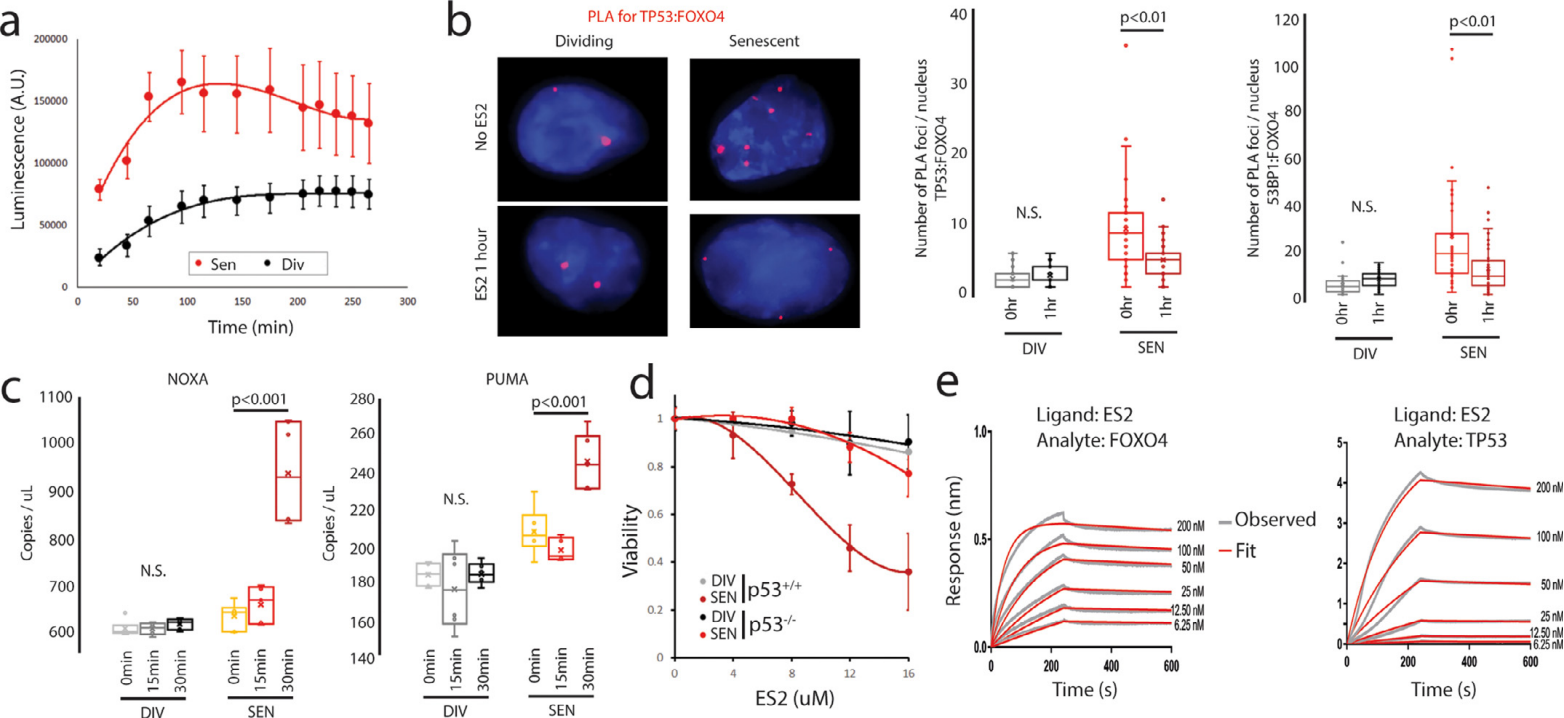
ES2 rapidly destroys TP53-FOXO4 foci leading to TP53 mediated apoptosis. **a,** Annexin V live staining showing that rapid induction of apoptosis is greater in senescent (Sen) compared to dividing (Div) cells (p=0.001) (n=5 for each time point). **b,** Proximity Ligation Assay showing increased FOXO4-TP53 and FOXO4-53BP1 foci (red) in senescent cells (n=41 for each) compared to dividing cells (n=41 and 36, respectively) (p<0.001). In addition, FOXO4-TP53 foci and FOXO4-53BP1 foci are reduced 1 hour after ES2 treatment in senescent cells (n=33 and 47 respectively) (p<0.01) but not dividing cells (n=41 and 44). **c,** ddPCR expression analysis shows that ES2 does not increase TP53 target genes in dividing cells, but shows a statistically significant increase after 30 minutes of ES2 treatment in senescent cells (n=6 for each time point) (p<0.001). **d,** Line plot of relative viability of MCF10a dividing (black) and senescent (red) cells with or without TP53 following a range of ES2 treatments. **e,** Bio-layer Interferometry analysis reveals ES2 preferentially binds FOXO4. Representative sensorgrams of FOXO4 binding to biotinylated ES2 peptide immobilized on streptavidin sensors. Representative sensorgrams of TP53 binding to biotinylated ES2 peptide immobilized on streptavidin sensors. See also Fig. S4 and S5.

First, we wanted to understand the kinetics of apoptosis using a live cell Annexin V stain. We found a rapid induction of apoptosis upon treatment with 8 µM ES2 in A375 cells (Fig. 7**a**). Annexin V staining increased within two hours of treatment, then plateaued, for both senescent and dividing cells. Senescent cells had a four-fold higher rate of apoptosis compared to dividing cells (p<0.001, linear regression comparison). Next, we tested if ES2 could interfere with direct binding of FOXO4 to TP53 using the proximity ligation assay. The proximity ligation assay measures if two proteins are within 40 nm of each other. Based upon the Annexin V data, we used the 1-hour post ES2 treatment to examine the effects of ES2 on FOXO4-TP53 foci. We found that ES2 treatment had no effect on FOXO4-TP53 foci in dividing cells, but reduced senescent cell foci by 2-fold (p<0.01) (Fig. 7**b**). We corroborated these results using 53BP1 antibody, which binds to TP53 and can be part of the same complex (Fig. 7**b**) and performing Co-IP (**Fig. S4**). Next, we tested if the released TP53 is responsible for the apoptotic signalling cascade by examining known downstream targets of TP53, *PMAIP1* (NOXA) and *BBC3* (PUMA) [63]. Dividing cells showed no response to ES2 treatment, but senescent cells showed a significant increase in levels 30 minutes after ES2 treatment for both NOXA and PUMA targets (p<0.001) (Fig. 7**c**). Finally, we showed that senescent *TP53* knockout MCF10a cells (p53^−/−^) were resistant to ES2 mediated cell death compared to senescent *TP53* wildtype MCF10a cells (p53^+/+^) (Fig. 7**d**). These results suggest that ES2 rapidly induces TP53 mediated apoptotic signalling cascade via the dissociation of FOXO4-TP53 foci.

Since our molecular modelling was designed for ES2 to preferentially bind to FOXO4 over TP53, we sought to experimentally characterize and validate the binding interactions of ES2 peptide for FOXO4 and TP53 using Bio-layer Interferometry (BLI) technology **(**Fig. 7**e,** Table 2.). Kinetic BLI experiments were performed using biotinylated ES2 peptide immobilized on a streptavidin sensor for binding to FOXO4, TP53 or a cytoplasmic protein, GFP **(**Fig. 7**e, S5)**. Both FOXO4 and TP53 bound ES2 peptide, while no binding was detected for GFP. Kinetic data were fit to a simple Langmuir 1:1 binding model to determine the kinetic rate constants and the binding affinities **(**Table 2**)**. FOXO4 binds ES2 rapidly with an association rate *k*_a_ of 1.17 × 10^5^ ± 3.07 × 10^2^ M^−1^ s^−1^ to form the ES2-FOXO4 complex, which slowly dissociates. Conversely, the ES2-TP53 complex forms three times more slowly with a *k*_a_ of 3.8 × 10^4^ ± 1.2 × 10^2^ M^−1^ s^−1^; however, shows a similar slow dissociation as the ES2-FOXO4 complex. Finally, the binding affinity (*K*_D_) of ES2 for FOXO4 and TP53 are 0.660 ± 0.006 nM and 2.3 ± 0.018 nM, respectively, revealing that FOXO4 binds ES2 with approximately 4-fold higher affinity compared to TP53. Finally, we experimentally determined the percentage of α-helix for ES2 to be 36% using circular dichroism, similar to the computational prediction of 34% (**Fig. S5**). Taken together these experimental results corroborate the computational, rational design of the ES2 peptide as having a preferential binding for FOXO4 compared to TP53.

**Table 2:** Kinetic parameters of FOXO4 and TP53 binding to ES2 peptide ligand at 30^ο^C. Kinetic rate constants were derived by fitting the experimental data to 1:1 Langmuir binding model in ForteBio Data Analysis HT 10.0 evaluation software. *k*_a_ is the association rate, *k*_d_ is the dissociation rate and *K*_D_ the equilibrium dissociation constant. For each analyte (FOXO4 or TP53), kinetic parameters were derived from a global analyses of six ES2 immobilized Streptavidin sensors each dipped into a different concentration of analyte ranging from 6.25-200 nM. NB – no binding.

Analyte	*k*_a_ (M s)^−1^	*k*_d_ (s^−1^)	*K*_D_ (nM)
FOXO4	1.2 × 10^5^ ± 3.1 × 10^2^	7.7 × 10^−5^ ± 7.2 × 10^−7^	0.66 ± 0.01
TP53	3.8 × 10^4^ ± 1.2 × 10^2^	8.7 × 10^−5^ ± 6.3 × 10^−7^	2.30 ± 0.02
GFP	NB	NB	NB

### 3.6 ES2 results in apoptosis and a survival advantage in different melanoma mouse models and senolysis in aged mice

To determine if ES2 could eliminate a developing cancer that is induced into senescence, we utilized two different mouse models: (i) a transgenic mouse model of melanoma, and (ii) an orthotopic xenograft mouse model. For the transgenic mouse model, the *Cre* gene is under the control of the Tyrosinase promoter, which restricts Cre expression to melanocytes [64]. In addition, the mouse contains conditional, floxed alleles for the oncogene, *Braf^V600E^*, and the tumour suppressor, *Pten*. In the Tyr-Cre mouse, Cre is fused to the oestrogen receptor, therefore only when treated with tamoxifen, will Cre result in BrafV600E activation and *Pten* loss. Only the combination of BrafV600E activation and *Pten^+/−^* or *Pten^−/−^* results in sporadic melanoma formation [65]. To induce senescence in these sporadic melanomas, we chose to deliver the Braf inhibitor, Dabrafenib. Dabrafenib is highly specific for BrafV600E over wild-type Braf and prevents growth of cells with the *Braf^V600E^* mutation [66] and Braf inhibition is known to cause premature senescence [67]. Above, we showed that the combination of Dabrafenib and ES2 results in increased apoptosis of the Braf-mutant cell line, A375, supporting that this combination could eliminate dividing cancer cells.

**Fig. 8:**
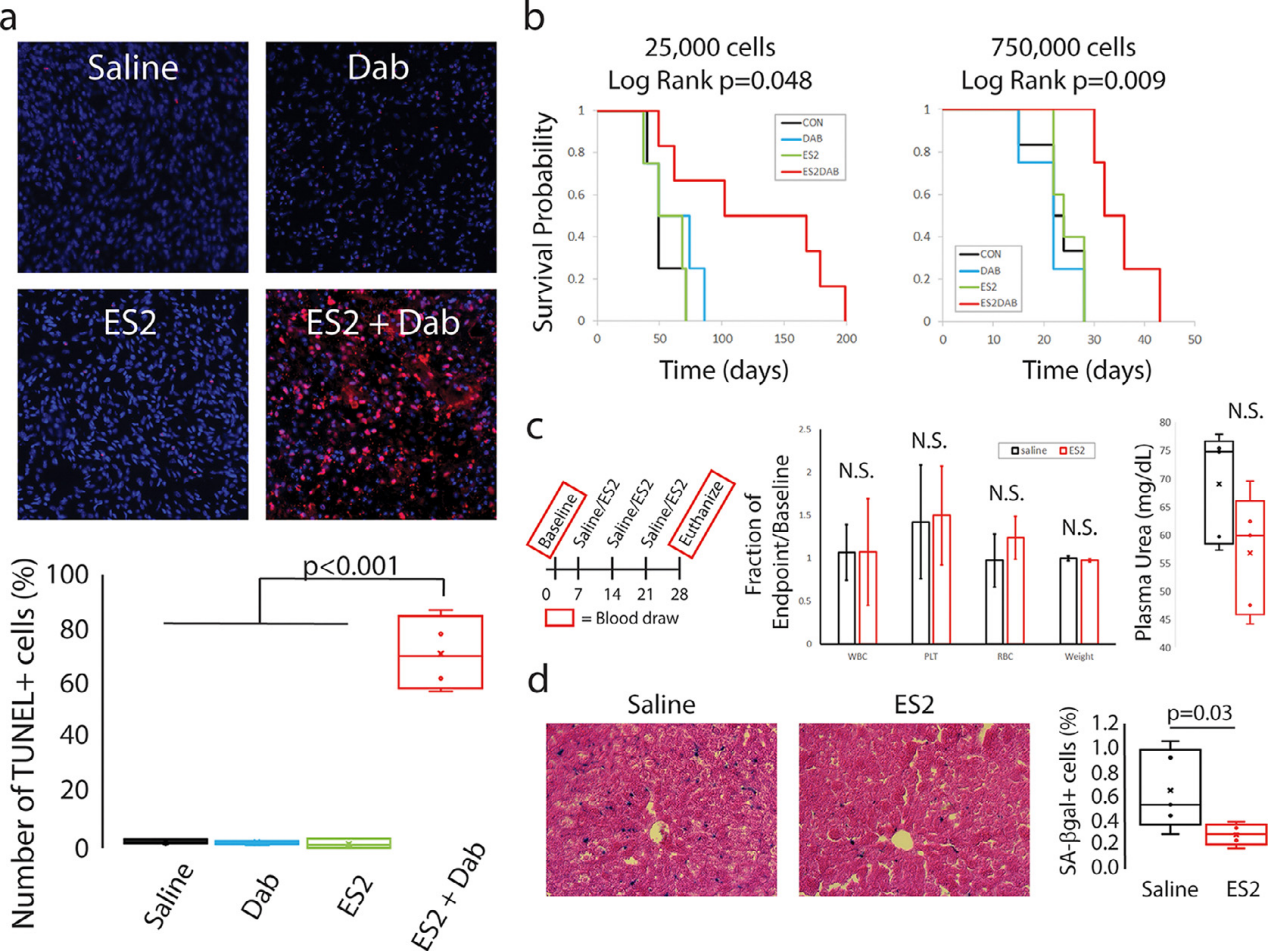
Concomitant treatment of ES2 plus dabrafenib results in apoptosis of melanoma cells. **a,** Images of melanoma sections from Tyr-BE transgenic mice. The tissue sections were stained for TUNEL+ cells (red) to measure apoptosis and DAPI (blue) for total nuclei. Quantification of the number of TUNEL+ cells in saline (n=4), Dabrafenib alone (n=4), ES2 alone (n=3), and ES2 + Dabrafenib (n=4) treatment. ES2 + Dab resulted in a 23-fold induction in apoptosis compared to saline, Dab alone or ES2 alone (p<0.001). There was no difference between saline, Dab alone or ES2 alone (p=0.99). ANOVA Scheffe PostHoc test. **b,** Concomitant treatment of ES2 plus dabrafenib improves overall survival. Kaplan-Meier curves for survival of mice under different treatments. CON – vehicle alone, ES2 – ES2 alone, DAB – Dabrafenib alone, ES2DAB – ES2 plus Dabrafenib. For 25,000 cells: CON – n=4, ES2 – n=4, DAB – n=4, ES2DAB – n=6. Log rank test of significance. For 750,000 cells: CON – n=6, ES2 – n=5, DAB – n=4, ES2DAB – n=4. **c,** Treatment regimen for aged mice. No statistically significant (N.S.) difference in change in White Blood Cells (WBC), Platelets (PLT), Red Blood Cells (RBC) or weight of mice between ES2 treatment and controls (n=5 per treatment). No statistically significant difference in endpoint plasma urea between ES2 treatment and controls (n=5 per treatment). **d,** Representative images of liver sections stained for SA-β-gal. Quantification of SA-β-gal+ cells in liver section shows reduced SA-β-gal+ cells after ES2 treatment (p=0.03) (n=5 per treatment). Student T-test. See also Fig. S6.

These transgenic mice, after treatment with 4-hydroxy-tamoxifen, sporadically developed melanomas (1-5 per mouse), typically on the back of the mouse within 3-5 months of age. Once the melanomas grew to 5-10 mm in size, each tumour was directly injected with either vehicle control, ES2, Dabrafenib, or ES2 plus Dabrafenib. Treatment was administered daily over two days and the melanomas were removed one day after the last treatment. The melanomas were then sectioned and stained for SA-β-gal and the apoptotic marker, TUNEL. Dabrafenib strongly induced senescence of the melanoma compared to vehicle treatment (**Fig. S6**) but had no effect on apoptosis (Fig. 8**a**). Importantly, the combination of ES2 plus Dabrafenib resulted in a 23-fold increase in apoptosis of cells within the melanoma (Fig. 8**a**) compared to all other treatment groups (p<0.001).

For the orthotopic xenograft mouse model, we injected mouse ears with either a high (750,000) or low ( 25,000) number of dividing A375 human melanoma cells. One day after injecting the cells, the ears were locally injected with either vehicle control, ES2, Dabrafenib, or ES2 plus Dabrafenib. Treatment was administered daily over two days and then the mice were followed for a survival study, where mice were euthanized when tumours reached 2 cm. Upon injection of the high number of cells, control mice develop local and distant tumours by about 3-4 weeks. Treatment with ES2 or Dabrafenib did not alter survival times. However, ES2 plus Dabrafenib increased survival times by 50% (p=0.009) (Fig. 8**b**). Upon injection of the low number of cells, control mice develop local and distant tumours after about 8 weeks. Again, ES2 or Dabrafenib had no effect on survival time. Interestingly, the combination treatment of ES2 plus Dabrafenib resulted in a greater than 65% increased survival time with 50% of mice surviving twice as long as untreated mice (p=0.048) (Fig. 8**b**). These results suggest that ES2 specifically eliminates senescent cancer cells and when used in combination therapy with a pro-senescence agent can slow cancer and improve survival.

Finally, we systemically delivered ES2 to aged mice (82 weeks) to determine the efficacy of ES2 in a model of ageing. ES2 was delivered to mice intravenously via tail vein for a total of 3 doses weekly. ES2 had non-significant changes on the numbers of white blood cells, platelets, or red blood cells over the four-week experiment. In addition, there was no significant change in the weight of the mice throughout the experiment. Interestingly, plasma urea levels, while not significant, were reduced in the mice treated with ES2 (Fig. 8**c**). These results all suggest that ES2 has minimal toxicity after multiple doses in aged mice. Finally, and most importantly, ES2 reduced the number of senescent cells in the liver by half as measured by SA-β-gal activity (p=0.03) (Fig. 8**d**). These results suggest that ES2 can eliminate senescent cells that accumulate with age via systemic delivery.

## 4 Discussion

Here we used a combination of molecular modelling, biochemistry, in vitro and in vivo experiments to specifically target FOXO4 in the FOXO4-TP53 interaction and show induction of apoptosis of senescent cancer cells. First, we showed that we can generate a series of peptides that have senolytic activity based on molecular modelling of the FOXO4-TP53 interaction. After evaluating the best candidate, we then showed that ES2 colocalized to FOXO4 foci and preferentially bound FOXO4 over TP53. While the typical measure for BLI is the dissociation constant, we found a rapid association of ES2 with FOXO4 suggesting that ES2 could prevent TP53 from ever binding to FOXO4 and thus allowing TP53 to carry out cellular apoptosis. Finally, we went on to show that combination therapy of ES2 and a Braf inhibitor results in apoptosis and a survival advantage in mouse models of Braf mutant melanoma and reduced senescent cells in ageing mice. While we focused mostly on senescent cancer cells in this study, our results suggest that ES2 is effective at eliminating both normal and cancer senescent cells.

The removal of senescent cells has been shown to improve age-related diseases including cancer in mouse models [7], [8], [9]. Cancer is a disease of ageing and there are numerous reasons why cancer is an intriguing target for senolytics. While senescence is a mechanism that cells can use to prevent neoplasia, the senescent cancer cells remain within the body leading to possibility of reactivation. There are several reasons that senescent cancer cell removal will be beneficial. First, the elimination of OIS cells could prevent these cells from escaping cell cycle arrest leading to tumour formation. Second, the elimination of senescent fibroblasts that block chemotherapeutic access to the cancer cells would allow for improved drug penetrance and therefore increase chemotherapeutic efficacy. Third, elimination of residual disease after chemotherapy would reduce the chance of recurrence and prevent unnecessary surgery that can destroy the normal tissue. Fourth, combination treatment of the senolytic with targeted, senescence-causing therapies would increase specificity and reduce off-target effects. Interestingly, we have shown that our rationally designed senolytic, ES2, can eliminate residual disease and work in combination with a pro-senescence therapy. However, there is no reason to doubt that ES2 can target other aspects of senescence in cancer. Finally, as cancers are detected and treated earlier, the quality of life may decline due to harsh effects of cancer treatments. It is conceivable this decline could be reversed or potentially eased by eradicating senescent cells with senolytic agents such as ES2, hence the therapeutic opportunities of senolytics in early cancer treatment deserves further investigation.

Many companies are beginning clinical trials to understand the role of senolytics in improving age-related diseases [68]. Multiple studies have shown the FOXO4-TP53 interaction to be important for the induction of senescence [16, 17, 69]. More recently, Baar *et al*. showed that a D-amino acid peptide derived from the N-terminus of the FH led to selective targeting of senescent cells through TP53 mediated apoptosis [17]. Their peptide, FOXO4-DRI, was selected from screening a small list of peptides derived from both FH and CR3 domains of FOXO4 and it was illustrated to bind TP53 [17]. Wang et al. was the first study to address the binding interactions between FOXO3 and TP53 [53]. They systematically evaluated the binding affinities of inter- or intra-domain complexes of FOXO3 and TP53, unveiling two strong interactions *(i)* an intramolecular interaction between the FH and CR3 of FOXO3 and *(ii)* an intermolecular one between the CR3 of FOXO3 and DBD of TP53. Since the domains of FOXO3 and FOXO4 show high sequence conservation [53], the FOXO3 findings by Wang et al. should be valid for FOXO4. Thus, targeting the CR3 domain may be better than the FH domain of FOXO4 for blocking the binding of TP53 to FOXO4.

To understand the role of the CR3 and FH domains of FOXO4, we have employed comprehensive computational analysis to unravel the molecular interactions between FOXO4 and TP53. Our resulting models suggested that the CR3 domain, but not the FH domain, of FOXO4 was a strong binder of TP53. More importantly, our models enabled us to propose a rationale for the senolytic action of FH-derived peptides. DRI or FH-derived peptides directly interact with the CR3 domain of FOXO4. This interaction sequesters CR3 from binding to DBD of TP53 and this sequestration or inhibition of CR3 unleashes TP53 to carry its apoptotic function. Thus, we propose that activation of TP53 by FH-derived peptides mainly occurs indirectly by inhibition of the TP53 inhibitor domain, CR3. Overall, taken together with previous efforts, these insights encouragingly designate FOXO4-TP53 as a noteworthy complex not only for drug discovery studies combatting age-related diseases but also for our understanding of interactions of transcription factors.

FOXO4DRI peptide has the same sequence as human FOXO4, only it is formed by D-amino acids in the retro-reversed sequence [17]; no other optimizations have been made to this sequence. In addition to DRI modification, it was fused to a 12 amino acid long CPP sequence, essentially to promote its intracellular delivery. However, aside from this principal role, structural analysis revealed that the fused CPP was shown to interact with CR3 and to contribute to binding free energy. When binding contribution of the CPP is ignored, the native FOXO4 sequence in the DRI isoform would in fact be lower than the CPP fused form (-106.90 *kcal.mol^−1^*). In other words, the bare DRI form would have a comparable binding free energy to the FH domain in the L-form (-55.97 *kcal.mol^−1^*). In line with this observation, binding free energy predictions indicated that the first designed L-peptide (E1) which differs from native FOXO4 sequence by 4 mutations, binds to CR3 more strongly than DRI (Table 1). Overall, these observations indicate that L- to D- isomerism of FH-domain derived peptides do not impose any significant improvement on binding. DRI modification is indeed a strategy to improve in vivo potency of peptides particularly by conferring resistance to proteolysis [70, 71]. Otherwise, DRI peptides may fail to replicate the same binding event with their L-isoforms, particularly if the parent peptide contains alpha-helices [72]. In line with this paradigm, our computational results show that DRI modification of FOXO4 peptide does not present any significant advantage for CR3 binding, rather it needs optimization for improved binding to its molecular target(s). Essentially, we showed that basic amino acids potentiate CR3 targeting. This observation prompted screening of a CPP library leading to identification of another TAT-based CPP (ES1) as a strong CR3 binder.

Binding affinity predictions suggested that E1 peptide has a comparable binding affinity with ES2r2, albeit in vitro analysis showed that E1 had much less activity than ES2r2. A similar contrast between computational predictions and experiments is present in Baar *et al.* who reported that L-form of FOXO4 peptide had much less senolytic activity than the FOXO4-DRI form [17]. However, our computations predicted that DRI and L-forms showed a comparable binding affinity towards CR3. These discrepancies between in silico and in vitro results could be a result of the fact that L-peptides are more prone to proteolysis compared to DRI forms. In fact a D-arginine amino acid is inserted to the N-terminus of the ES2r2 to confer stability to L-peptides [73, 74]. Hence, despite comparable CR3 binding affinity, higher stability of ES2r2 than E1 due to the addition D-arginine would lead to such distinct in vitro activities. Similarly, given that DRI modification confers stability, the higher in vitro potency of DRI compared to L-form as observed in the study of Baar *et al.* [17] could be explained by DRI form's higher stability. Overall, we have shown that we can use in silico molecular modelling to generate a series of senolytic peptides targeted to the FOXO4-TP53 interaction. In the future, it would be beneficial to optimize theses peptides and generate small molecules that can improve upon the design of our senolytic peptides.

## Contributors

**Conception and Design:** Y. Ahiska, U. Sezerman, E. Timucin, J.M. Fischer

**Molecular Modelling:** S.S. Cinaroglu, B. Duan Sahbaz, E.S. Ozdemir, U. Sezerman, G. Bayram Akcapinar, E. Timucin

**In vitro Data Acquisition:** H.H. Le, A. Ors, M.M. Gomes, T.E. Kawashima, A. Quentel, J.S. Plaut, C.A. Origel Marmolejo, K. Bonic, J.C. Saldivar, J.M. Fischer

**In vivo Data Acquisition:** H.H. Le, E.C. Manalo, T.E. Kawashima, A. Quentel, J.M. Fischer

**Analysis and interpretation of data:** H.H. Le, S.S. Cinaroglu, E.C. Manalo, A. Ors, M.M. Gomes, J.S. Plaut, C.A. Origel Marmolejo, E.S. Ozdemir, G. Bayram Akcapinar, J.C. Saldivar, E. Timucin, J.M. Fischer

**Writing, review of manuscript:** H.H. Le, S.S. Cinaroglu, A. Ors, M.M. Gomes, J.S. Plaut, E.S. Ozdemir, J.C. Saldivar, S.V. Malhotra, E. Timucin, J.M. Fischer

**Study supervision:** E. Timucin, J.M. Fischer

## Declaration of Competing Interest

S.S. Cinaroglu, Y. Ahiska, U. Sezerman, G. Bayram Akcapinar, E. Timucin have a patent for the ES2 structure and are members of a company, Eternans Ltd. that is working on ES2 for therapeutic use. No potential conflicts of interest were disclosed by the other authors.

## Appendix Supplementary materials

Image, application 1
