# Supplementary material for: Molecular modelling of the FOXO4-TP53 interaction to design senolytic peptides for the elimination of senescent cancer cells
Source: eBioMedicine. 2021 Oct 21;73:103646. doi: 10.1016/j.ebiom.2021.103646 (PMC8546421; doi:10.1016/j.ebiom.2021.103646)
Supplement: Supplementary file 1 [file mmc1.docx]

**Table S1.** Peptide list.

| **Name** | **Sequence (H-..-OH)** | **Size (u)** | **pI** |
| --- | --- | --- | --- |
| Foxo4DRI *^*^* | TLRKEPASEIAQSILEAYSQNGWANRRSGGKRP*PPRRRQRRKKRG* | 5244.97 | 12.01 |
| E1 | *GRKKRRQRRRPP*PRKGGSRR**R**AWGNQ**R**YA**R** LI**R**QAIE SAPEKRLTLA | 5636.58 | 12.40 |
| ES2 | PRKGG**R**RR**R**AWG**RR RRRRRRRRRRRR**APRKRLTLA | 4646.56 | 13.30 |
| ES2r1 | PRKGG**R**RR**R**AWG**RR RRRRR**L **RRR**L**RR**APRKRLTLA | 4560.51 | 13.26 |
| ES2r2 | PRKGGSRR**R**AWG**RR R**Y **RRR**L **RRR**L**RR**APRKRLTLA | 4498.39 | 12.88 |
| ES1 | *YGRKKRRQRRRYGRKKRRQRRRYGRKKRRQRRR* | 4643.51 | 12.55 |
| The mutations to the native FOXO4 sequence were given in **bold** and the fused HIV-TAT in *italic*.  *^*^* All D-amino acids. | | | |

**Table S2.** Screening results.

| **Sequence (H-..-OH)** | ${\boldsymbol{\Delta}\boldsymbol{G}}_{\boldsymbol{bind}}$ **(*kcal.mol^-1^*)^*^** |
| --- | --- |
| YGRKKRRQRRRYGRKKRRQRRRYGRKKRRQRRR | -149.15 ± 8.05 |
| RRRRRRRRRRRRRRRRRR | -128.07 ± 10.47 |
| HHHHHHHHHHHHHHHHRRRRRRRRRRRRRRR | -123.06 ± 8.7 |
| RRRRRRRRRRRRRRRR | -114.74 ± 5.16 |
| TRQARRNRRRRWRERQR | -105.97 ± 6.47 |
| RRRRRRRRRRRRRR | -105.74 ± 6.68 |
| RRRRRRRRRRR | -104.94 ± 6.05 |
| RRRRRRRRRRRRRRR | -92.47 ± 5.98 |
| RRRRRRRR | -88.71 ± 8.61 |
| ARRRRCSGSGSGCGSGSGSCGRRRR | -88.05 ± 3.86 |
| RRRRRRRRRRRRGC | -81.9 ± 8.83 |
| RRRRRRRRR | -81.34 ± 5.75 |
| ACGRGRGRCRGRGRGCG | -79.23 ± 4.17 |
| SGRGKQGGKARAKAKTRSSRAGLQFPVGRVHRLLRKG | -77.73 ± 9.19 |
| YGRRRRRRRRR | -71.52 ± 5.81 |
| GGRRARRRRRR | -68.87 ± 8.78 |
| RLLRLLRRLLRLLRRLLRC | -66.88 ± 5.83 |
| YGRRARRRRRR | -65.69 ± 9.64 |
| RQIRIWFQNRRMRWRR | -65.43 ± 6.01 |
| RRRRR | -63.74 ± 15.81 |
| RNRSRRRRNRSRRR | -61.81 ± 8.22 |
| LGISYGRKKRRQRRRPPQ | -60.5 ± 10.54 |
| RRRRRR | -53.08 ± 7.56 |
| **^*^** ${\boldsymbol{\Delta}\boldsymbol{G}}_{\boldsymbol{bind}}$ was given as “mean±SD*”* of 10 calculations corresponding to 10 distinct CR3 conformers. The first three showed significantly higher binding free energy to CR3 than DRI (p < 0.001). | |


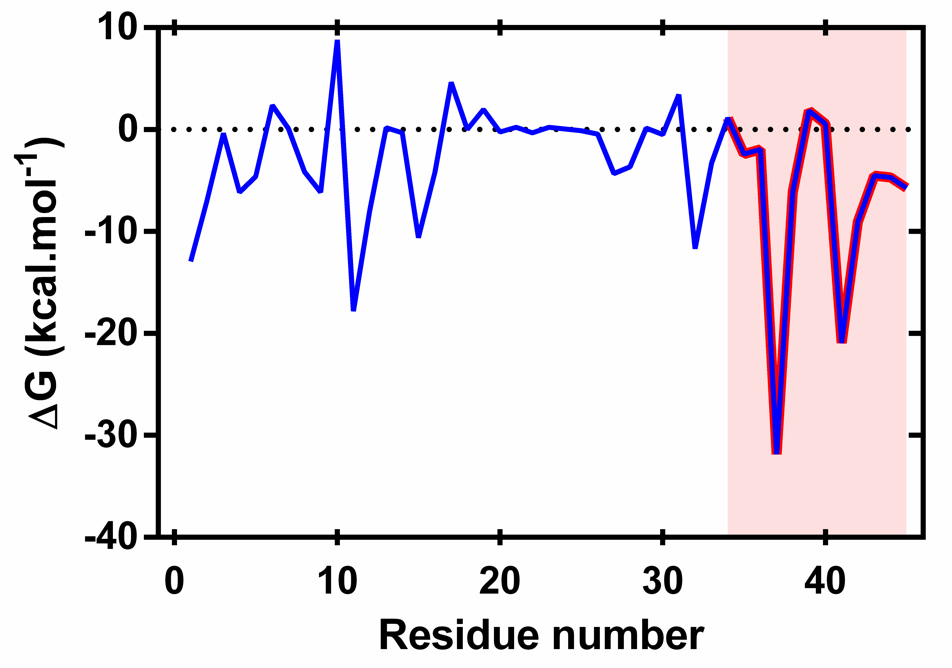


**Figure S1.** Binding free energy contribution of each amino acid from Foxo4-DRI. The region corresponding to the HIV-TAT fusion was shaded.





**Figure S2.** Mean difference plot of the post hoc comparisons that were conducted by the Tukey HSD test. Error bars mark the upper and lower limits of the 95% confidence interval of the mean difference (n=10).


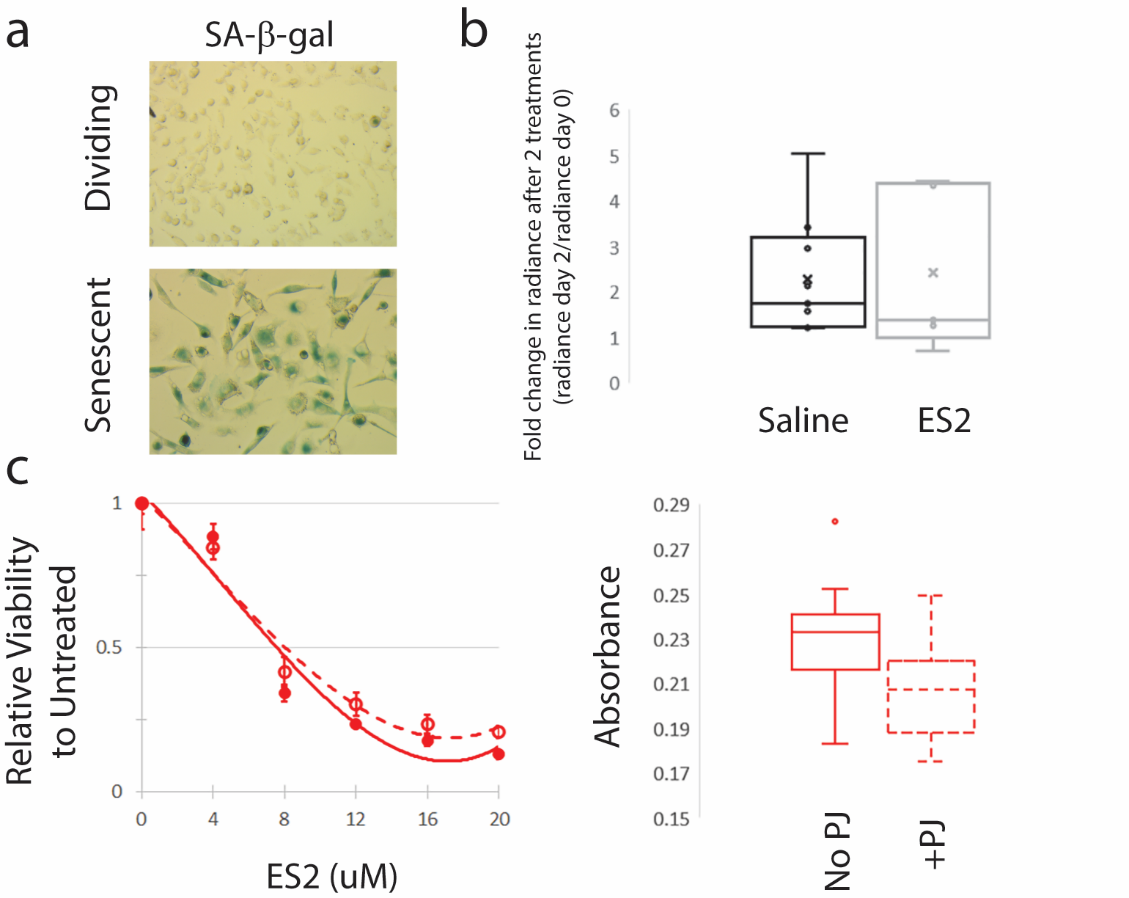


**Figure S3.** **a,** Representative images of dividing and senescent A375 cells stained for SA-β-gal. **b,** ES2 alone has no effect on dividing cancer cells. Dividing A375 cells were injected orthotopically and then locally injected twice with either saline (n=9) or ES2 (n=5) and then imaged one day later. There is no difference in gain of radiance between saline or ES2 treatments (p=0.87). **c,** ProteoJuice (PJ) was used to negate the effect of ES2’s positive charge on apoptosis. Encapsulating ES2 in PJ (dashed line) had no effect on viability in senescent cells compared to ES2 alone (solid line) (n=5 for each time point). PJ alone (dashed line) only reduced viability of senescent cells by 11% compared to no PJ (solid line).


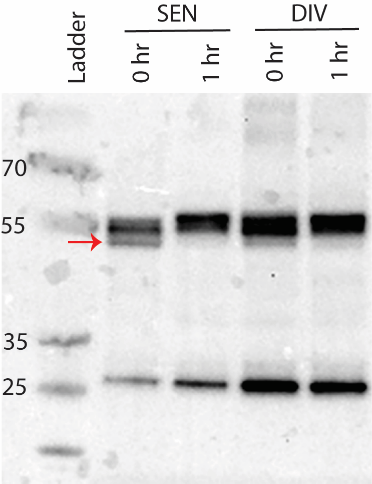


**Figure S4.** Co-immunoprecipitation of FOXO4 and TP53. Nuclear proteins were extracted from cells expressing FOXO4 protein tagged with Myc. Myc was pulled down and the subsequent blot was stained for TP53. Senescent (SEN) or Dividing (DIV) cells were treated with ES2 for 0 hours or 1 hour. The red arrow points out a band at 53kDa that is eliminated after treating with ES2.


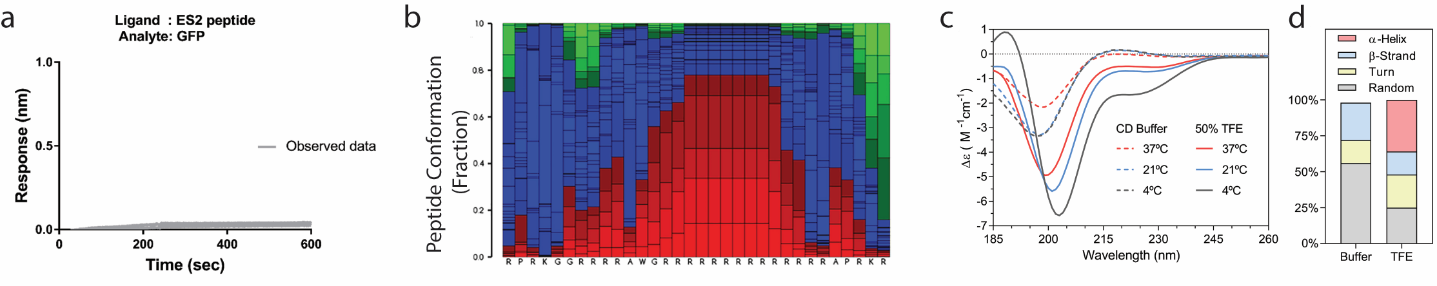


**Figure S5.** **a,** Bio-layer Interferometry analysis of ES2 peptide binding GFP. Biotinylated ES2 peptide immobilized on streptavidin sensors was allowed to bind six, 2-fold dilutions of the cytoplasmic protein GFP starting at 200nM. As expected, minimal binding of GFP (grey curves) for ES2 was observed even at the highest concentration of GFP. **b,** Peptide structure prediction profile of ES2 given as probabilities of secondary structure. helix – red (11/32 = 34%), coil – blue (19/32 = 59%), extended – green (2/32 = 6%). **c**, CD spectra of ES2 in dilute CD buffer with and without 50% TFE shows propensity of ES2 to form alpha-helices. **d**, Results of secondary structure analysis of ES2 CD spectra at 4ºC with or without 50% TFE. Estimations of the percent content of alpha-helix, beta-strand, beta-turn, and random coil structural elements are shown.


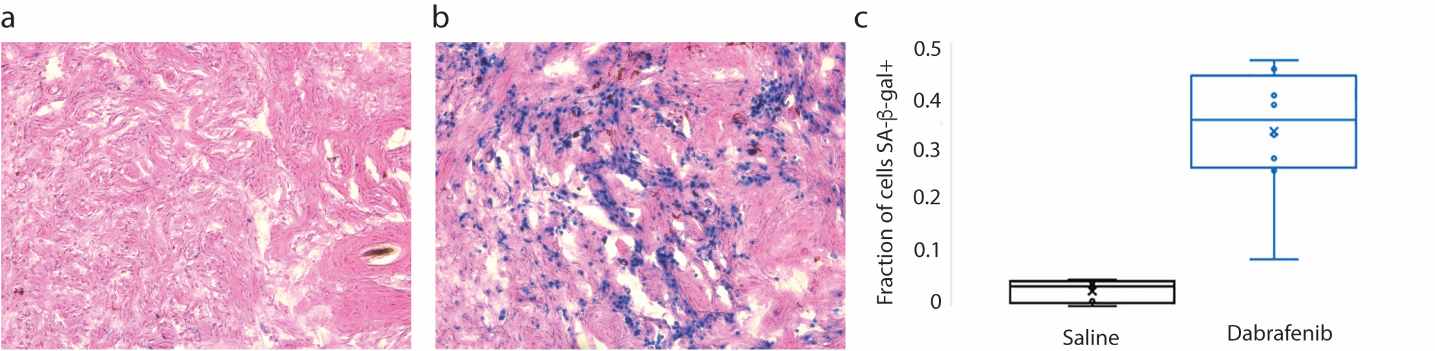


**Figure S6.** Fraction of senescent associated (SA) β-gal+ cells after dabrafenib treatment of melanomas. **a and** **b,** Images of SA-β-gal staining in saline treated **a**, and dabrafenib treated **b**, melanomas. **c,** Dabrafenib treatment (n=8) increases the fraction of SA-β-gal+ cells within the melanoma by 11-fold over saline treatment (n=5) (p<0.001).
